# Qualitative Versus Quantitative Mammographic Breast Density Assessment: Applications for the US and Abroad

**DOI:** 10.3390/diagnostics7020030

**Published:** 2017-05-31

**Authors:** Stamatia Destounis, Andrea Arieno, Renee Morgan, Christina Roberts, Ariane Chan

**Affiliations:** 1 Elizabeth Wende Breast Care, LLC. Rochester, NY 14620, USA; aarieno@ewbc.com (A.A.); rmorgan@ewbc.com (R.M.); 2 Volpara Solutions Limited, Wellington 6011, New Zealand; christina.roberts@volparasolutions.com (C.R.); ariane.chan@volparasolutions.com (A.C.)

**Keywords:** breast imaging, breast density, quantitative assessment, qualitative assessment

## Abstract

Mammographic breast density (MBD) has been proven to be an important risk factor for breast cancer and an important determinant of mammographic screening performance. The measurement of density has changed dramatically since its inception. Initial qualitative measurement methods have been found to have limited consistency between readers, and in regards to breast cancer risk. Following the introduction of full-field digital mammography, more sophisticated measurement methodology is now possible. Automated computer-based density measurements can provide consistent, reproducible, and objective results. In this review paper, we describe various methods currently available to assess MBD, and provide a discussion on the clinical utility of such methods for breast cancer screening.

## 1. Introduction

Mammographic breast density (MBD) describes the proportion of radiologically dense fibroglandular tissue in the breast. Dense tissue comprises the functional glandular tissue (epithelial cells of the mammary lobular and ductal system) and the fibrous stromal tissue (including collagen, blood vessels and immune cells) of the breast [1]. Due to their different attenuation properties, higher attenuating fibroglandular tissue appears white on a mammogram, as opposed to adipose or fatty tissue, which appears dark. Women exhibit a natural continuum of MBD, which is influenced by numerous factors, including age, ethnicity, endogenous and exogenous hormones, menopausal status, body mass index (BMI) and parity. MBD also has a high heritability, with twin studies estimating that approximately 60% of the variation in MBD is genetically determined [2,3,4].

MBD is an important consideration for both breast cancer screening and prevention. Due to their similar X-ray attenuation properties, dense tissue and tumors both appear white on a mammogram and numerous studies have shown the potential masking risk of MBD for cancer detection and negative association of increasing MBD with mammographic sensitivity [5,6,7,8,9,10,11]. Although the molecular and biological mechanisms underpinning breast cancer development are still being elucidated, MBD has also been established as a strong, independent risk factor for de novo development of breast cancer and for cancer recurrence [12,13]. Thus, MBD assessment has clinical utility for identifying women at increased risk of developing breast cancer and/or having reduced mammographic sensitivity, who might benefit from supplementary screening methods, preventative therapies, or even genetic analysis. It is, therefore, highly imperative that accurate and reliable measurements of MBD be used clinically, and much progress is being made in this regard. In this review, we will discuss the available methods of MBD assessment, their advantages and limitations, and MBD in the context of current clinical practice in the United States (US) and internationally.

## 2. Mammographic Density Assessment Methods

**Figure 1:**
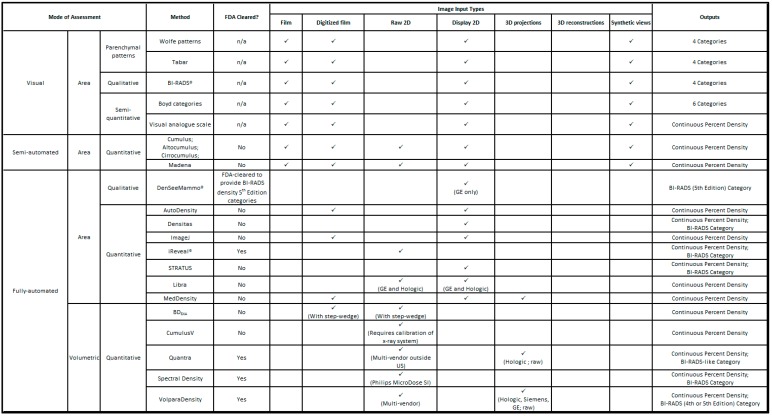
Mammographic Breast Density (MBD) Assessment Methods. Check marks denote that the method runs on the specified image input type; n/a: not applicable.

Given that a mammographic image is a 2-dimensional (area-based) representation of a 3-dimensional (volumetric) physiological phenomenon, numerous density assessment methods have been proposed and developed over the past 4 decades that measure various aspects of fibroglandular tissue [14,15,16,17]. These methods can be broadly classified by: (1) their mode of assessment (visual, semi-automated, fully-automated); (2) whether they measure area-based or volumetric parameters; and (3) whether they are qualitative or quantitative in nature (Figure 1). Many of the computerized methods are for research-only purposes although several of them are now commercially available for certain markets.

## 3. Visual Methods

### 3.1. Parenchymal Patterns

The concept of MBD as a risk factor for breast cancer was first proposed by John Wolfe in 1976 [18,19]. Wolfe described the relationship of a prominent duct pattern and breast cancer, leading to the hypothesis that if a prominent duct pattern was seen more frequently in women with breast cancer, then a prominent duct pattern may precede the development of breast cancer. “Wolfe’s classification” described four qualitative categories based on parenchymal patterns: N1 (“normal”) which constituted a breast made entirely of fat; P1 (“prominent 1”)—composed mostly of fat, but displaying prominent ducts behind the areola or in the upper axillary quadrant occupying no more than 25% of the breast; P2 (“prominent 2”)—displaying a more prominent duct pattern than P1 (often in a triangular pattern in the center of the breast), with a quarter or more of the breast being occupied; DY (“dysplasia”)—a general increase in breast density, with a possible minor involvement of prominent ducts. N1 and P1 have been determined as presenting lower cancer risk, while P2 and DY are higher-risk patterns. Additionally, Wolfe created a QDY (“quasidysplasia”) category for women below age 40–45 because while these women tend to display the DY pattern due to their young age, it is likely to regress to a lower-risk pattern after menopause.

An alternate pattern-based qualitative description system of breast density was developed by Lázló Tabár in 1997 [20]. This model of density assessment was based on a mixture of four mammographic building blocks making up the normal breast anatomy. These include nodular densities corresponding to the terminal duct lobular units; linear structures which correspond to either ducts or fibrous or blood vessels; homogeneous structureless densities which correspond to fibrous density; and radiolucent areas which correspond to adipose fatty tissue. Pattern I is characterized by predominantly dense tissue with nodular densities, with regions of fatty tissue. Pattern II indicates completely fatty breasts. Pattern III describes a mostly fatty breast with visible ducts behind the areola. Pattern IV are predominantly dense breasts with linear and nodular densities. Finally, pattern V comprises of high levels of homogenous density. Like the Wolfe patterns, Tabár patterns correspond to different risk levels. Patterns II and III (roughly corresponding to N1 and P1) represent fatty breasts and low risk of cancer. Pattern I is also considered “low cancer risk” as a breast of this density would still reveal pathological changes (it corresponds to Wolfe QDY). Patterns IV and V (corresponding to P1 and DY) describe dense breasts carrying high risk.

### 3.2. Semi-Quantitative

Pattern-based assessment has suffered from a lack of reproducibility [21,22,23,24,25,26,27]. To reduce the heterogeneity in the risk estimates, Norman Boyd et al. were the first to attempt to semi-quantify density visually using a six-category classification (SCC) [28]. This was the first method to transition away from describing patterns of tissue density to a more objective assessment using percentages. The SCC is a quantitative area-based measure that consists of visual estimates of density utilizing a thresholding method; these being A (0% density), B (0 to <10%), C (10% to <25%), D (25% to <50%), E (50% to <75%) and F (≥75%). Increasing SCC categories were found to be positively associated with increased breast cancer risk [29]; though to a lower magnitude than the initial Wolfe estimates. While useful for epidemiological studies, the SCC has not been routinely used in clinical practice.

Another semi-quantitative approach involves a visual estimation of the breast density using a Visual Analogue Scale (VAS). Readers mark along a continuous scale that represents 0–100% density, and these score sheets can then be scanned through software to obtain the percent breast density.

VAS has been used in large clinical studies [30,31,32,33,34] and is considered preferable to some of the thresholding-based methods (described below) as it is less laborious and does not require specific reader training.

### 3.3. BI-RADS

Another visual method is the Breast Imaging-Reporting and Data System (BI-RADS) developed by the American College of Radiology (ACR), which is intended to provide a standardized method for reporting and streamlining imaging interpretations. This method also sought to help indicate the potential masking effect of dense breast tissue. Although a qualitative system to start with, the 4th edition of BI-RADS incorporated a quantitative component to the category definitions [35]. More recently, the ACR has released the BI-RADS 5th Edition [36], which has removed the quantitative aspect to return to a more subjective scoring system (Table 1). Both in the US and internationally, the BI-RADS density classification system is the most widely used clinically by radiologists.

## 4. Semi-Automated Density Assessment

Considering the limitations of visual density assessment, less subjective and more quantitative measures have been sought. Martin Yaffe and researchers from the University of Toronto developed Cumulus as a semi-automated computerized measure of dense area [37]. Cumulus uses reader-based thresholds to define the breast edge and regions of density on a digital or digitized mammogram. Each pixel within the breast area between the skin line and pectoral muscle is segmented into either fat or fibroglandular tissue; this defines the cut-off point. Cumulus has been the gold standard for quantitative density measurement for many years now; several validation studies have demonstrated this method’s high reproducibility [6,38,39]. Strong positive intra-user and inter-user correlations have been noted [16]. Positive results have been reported in evaluation of Cumulus with digital breast tomosynthesis (DBT) in the US; however, the software has been shown to overestimate breast density by 3% with DBT in comparison to digital mammography [40]. Madena is another threshold-based method for density measurement [41]. However, these methods require training and human input to define the threshold of density, which introduces subjectivity, potentially limiting their widespread clinical use (both Cumulus and Madena are for research purposes only). More recent work by researchers at the University of Melbourne are investigating what the impact of altering the cut-off or threshold level is on breast density assessment. For example, their Altocumulus and Cirrocumulus methods, which utilize full-field digital mammography (FFDM) images, use increasingly higher thresholds to define what is dense and non-dense tissue compared to Cumulus [42].

## 5. Fully-Automated Density Assessment

Several fully-automated breast density methods have been developed to provide objective measures of MBD that can be more easily integrated into clinical practice. However, as noted below, these methods vary widely in their approaches and which parameters of fibroglandular tissue are being measured.

### 5.1. Area Methods

Many groups have developed automated area-based methods of MBD assessment, effectively removing the human interactive component of the Cumulus and Madena methods. Several of the research-only tools include: (1) an ImageJ based method developed at Karolinska Institute [43]; (2) AutoDensity, developed by the University of Melbourne; (3) LIBRA, a method based on multi-cluster fuzzy c-means segmentation developed at University of Pennsylvania [44]; (4) STRATUS, a machine learning approach developed by the Karolinska Institute [45], that also provides a computerized BI-RADS score based on cut-offs of their percent density; and (5) MedDensity, an automated algorithm based on maximum entropy thresholding, developed at University of Genova [46]. These methods work with images from a range of modalities, including film screen, digital mammography and DBT.

iReveal^®^ [47] (formerly known as M-Vu Breast Density) and Densitas (DM-Density) are commercially-available automated algorithms (Densitas is currently only cleared in Canadian and European markets) that compute area density [48] and then classify density into BI-RADS-analogous categories; however, independent research using iReveal or Densitas has been limited [49,50,51]. DenSeeMammo recently received Food and Drug Administration (FDA) clearance to provide density categories based on the BI-RADS 5th edition definitions. In contrast to STRATUS, iReveal and Densitas, DenSeeMammo do not output a quantitative measure of breast density, and rely on a nearest neighbor approach using a reference database to determine the BI-RADS category.

### 5.2. Volumetric Methods

By taking advantage of the valuable information contained in the raw mammographic image, several approaches have been developed that estimate the actual volume of fibroglandular tissue in the breast. In contrast to the area-based methods described above, volumetric methods are more anatomically relevant, as they take the depth of fibroglandular tissue into consideration.

BD_SXA_ and Cumulus V are two research tools that estimate volumetric breast density in a fully-automated fashion. Researchers at the University of California, San Francisco developed the BD_SXA_ method based on Single X-ray Absorptiometry techniques [52]. The BD_SXA_ method requires that mammograms are taken with a phantom step-wedge included on each image. The step-wedge is compressed to the same thickness as the breast, and is comprised of gray-scale references of dense and non-dense tissue that can be compared to the pixel values in the mammogram to determine the MBD. An extension of the Cumulus algorithm, CumulusV, estimates volumetric breast density from the breast thickness and X-ray attenuation, after correction for potential errors in the readout thickness of the mammography system [53,54]. For each mammography system, however, prior calibration (using breast-equivalent phantoms) of the X-ray attenuation of breast tissue as a function of thickness and composition is required.

In 2008, Quantra was the first fully-automated volumetric MBD tool to become commercially available. Quantra is a volumetric breast density (VBD) assessment tool produced by Hologic (Hologic Inc., Bedford, MA, USA; [55]) and was based on research carried out at the University of Oxford by Ralph Highnam and Michael Brady. Van Engeland published on implementing this method for raw digital mammograms [56]. Highnam and Brady continued development of their algorithm, and gained FDA clearance for VolparaDensity (Volpara) in 2012 (Volpara Health Technologies, Wellington, New Zealand; [57]. Using the pixel intensities in the raw mammographic image and known X-ray attenuations of adipose versus fibroglandular tissue, Quantra and Volpara estimate the thickness of adipose versus fibroglandular tissue for each tissue “column”. The tissue columns are then summed to obtain the total breast volume, volume of fibroglandular tissue and their ratio (expressed as a percentage), VBD. The general concepts underlying each algorithm are similar, although there are some notable differences. Quantra uses an absolute physics model, in contrast to the relative physics approach used by Volpara, which finds in each image, a pixel signal corresponding to purely adipose tissue that is used as an internal reference [57,58]. Pixels that are deemed to correspond to significant amounts of dense tissue are also used to determine an area-based estimate of MBD by Quantra.

Volpara also outputs Volpara Density Grades (VDG) based on preset VBD thresholds, which are analogous to the BI-RADS visual density categories. Based on validation work compared to a panel of Mammography Quality Standards Act (MQSA) radiologists, the software can be configured to provide VDG scores that align with either the 4th or 5th edition BI-RADS definitions. Quantra 3 version 2.2.1 also outputs a BI-RADS-like score, analogous to the BI-RADS 5th edition density categories, by mapping an estimate of area-based density to BI-RADS. Several studies have compared the agreement between radiologists’ density assessments and the BI-RADS-like categories output from these software methods. Gweon et al. of South Korea compared Volpara (version 1.5.1) to BI-RADS categories as determined by radiologists utilizing FFDM images, and revealed a positive correlation between BI-RADS categories and the automated density assessment [59]. The study also reported moderate to substantial inter-observer agreement with the use of BI-RADS 4th edition density categories. Similarly, Seo and colleagues from the Sungkyunkwan University School of Medicine also compared Volpara automated measurement (version 1.4) with visual BI-RADS assessment (also 4th edition), utilizing FFDM images. VDG showed good agreement with visual assessment [60]. The authors did conclude, however, that Volpara could be less reliable in breasts with scattered density. Additionally, it was noted that differences between automated and visual assessment can be affected by physical factors of the mammography system. Brandt et al. compared both Volpara (version 1.5.0) and Quantra (version 2.0) readings for 6081 women undergoing mammography at the Mayo Clinic or within the San Francisco Mammography Registry to radiologist-assigned BI-RADS (4th edition) [61]. Both automated methods displayed moderate agreement to radiologists, with Quantra having a weighted kappa value of 0.46 and Volpara of 0.57.

A 2013 paper compared BD_SXA_, Volpara (version 1.4.3) and Quantra (version 3.2) to breast magnetic resonance imaging (MRI), which is considered the “ground truth” for measuring the accuracy of VBD estimates [62]. Volpara showed the highest correlation to MRI for dense volume and BD_SXA_ showed the highest correlation for VBD, though it is important to note that the slopes were substantially different from the identity line (1.0) for percent fibroglandular volume to MRI. Gubern-Merida similarly evaluated VDG assessment on FFDM mammograms by comparing results to volume estimates obtained from MRI data. Utilizing Volpara (version 1.4.3), high correlation to MRI was found for volumetric measurements from FFDM [63].

Philips Spectral Density Measurement Tool is another volumetric assessment method, but one that is based on spectral breast density as opposed to X-ray contrast [64]. It is a component of the Philips MicroDose mammography system and relies on dual energy decomposition to measure the amount of adipose and fibroglandular tissue in the breast. A photon counting detector uses energy thresholds to sort photons into high and low energy. The Philips Spectral Density Measurement Tool outputs VBD, total fibroglandular volume and total breast volume as well as a “MicroDose Density Score”, analogous to a BI-RADS categorization [65]. However, clinical studies using this method have been small and limited [66,67,68].

## 6. Advantages and Limitations of MBD Assessment Methods

### 6.1. Area vs. Volumetric MBD

**Figure 2:**
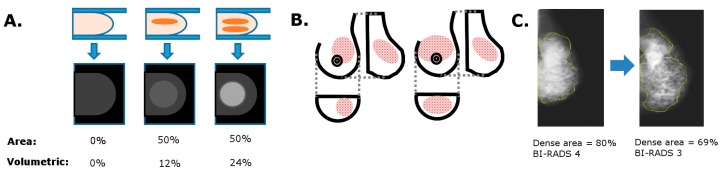
Limitations of area-based density measures. (**A**) Area-based density cannot accurately measure regions of overlapping tissue or increased tissue depths; (**B**) Areas of density that appear different on one view (e.g., CC (craniocaudal)) can appear similar on another view (e.g., MLO (mediolateral oblique)); (**C**) 1 cm of extra compression can alter the visually assessed density and thus BI-RADS category.

Due to their 2-dimensional nature, area-based methods of MBD measurement inherently suffer from a set of biases. Firstly, they cannot determine the depth of the dense tissue or overlapping regions of dense tissue in the breast (Figure 2A). They also do not consider the fact that the same amount of dense tissue can appear markedly different on one view compared to another (e.g., craniocaudal vs mediolateral oblique) (Figure 2B). Increased or decreased compression on the same breast can spread the tissue to different extents, and this can alter the apparent area-based density without changing the true amount of dense tissue in the breast (Figure 2C). Estimating the actual volume of dense tissue would lead to a more accurate reflection of breast anatomy, although it is still under debate as to which parameters may be more useful for certain applications such as breast cancer risk assessment.

### 6.2. Consistency in MBD Measurements

As described in later sections, there is a growing demand for a reliable and consistent method of assessing density. Breast imagers have been using visual assessment for over a decade. While the visual method of density assessment is well-established, both in widescale acceptance and the relation of risk of interval cancer, as discussed in a later section, they do have limitations. This assessment method relies on human judgement and is thus inherently subjective. Individual radiologists can show high consistency, as determined by intra-reader agreement on density reading studies in both the US and abroad [69,70,71,72]. However, two recent studies have highlighted the large variability that can exist between observers. Sprague et al., as part of the PROSPR consortium, compared BI-RADS 4th Edition density readings from 83 radiologists from three health networks and the percentage of women deemed “dense” (BI-RADS 3 or 4) was calculated [73]. The median percentage of mammograms judged as “dense” was 38.7%, but the overall range showed substantial variation, going from 6.3% to 84.5%. Furthermore, of the women whose density was re-assessed by the same radiologist after an average period of 1.2 years, 10% changed in major density classification (going either from “non-dense” to “dense”, or vice versa). However, when it was a different radiologist performing the assessment, 17.2% of women received a different classification—a 72% increase in discrepancy, even though the period between mammograms remained the same. This indicates that a woman’s visual density assessment can be highly dependent on the reader. Another study by Irshad et al. in the US compared the effect of changing from 4th to 5th edition of BI-RADS [70]. The study revealed that both intra- and inter-reader agreement on density ratings decreased significantly when changing to the new system. Inter-reader agreement reduced from “good” to “moderate”, as assessed by Fleiss-Cohen weighted kappa (0.65 to 0.57). However, contradicting these studies was a recent publication from Raza et al. out of Brigham and Women’s Hospital. The study investigated the accuracy of visual mammographic density assessment in relation to training, to determine if training can improve assessment [74]. Results demonstrated that training positively impacted the accuracy of readers’ breast density assessments, with increase from 65% before training to 72% after training. Study authors also evaluated agreement between qualitative and quantitative density assessment methods, and found substantial agreement between the two (κ = 0.78).

The 5th edition BI-RADS criteria are more subjective and the new classification may see greater variation being introduced into density assessment. The new BI-RADS notes that even in breasts where <50% of the volume of the breast is dense, if the fibroglandular tissue is “sufficiently dense to obscure small masses” then the breast should be classified as “Heterogeneously Dense”; however, the visual interpretation of what is considered to be “sufficiently dense” can be subjective. Other MBD measures that report a BI-RADS analogous category, whether they be area-based or volumetric, will also need to address how exactly they will determine what “sufficiently dense” is from a quantitative stand-point. When comparing automated methods against each other, as was done in a 2015 study by Brandt et al. [61], variation can be seen. The study included FFDM mammography examinations from Mayo Clinic or one of four sites within the San Francisco Mammography Registry. The study found moderate agreement when comparing visual BI-RADS assessment, Volpara and Quantra, but found differences of up to 14% in dense tissue classification. This highlights a chief factor to consider, as accurate identification of patients with dense breast tissue is important, and variations in dense tissue classification could potentially substantially affect clinical decision making.

A recent study out of South Korea evaluated automated volumetric measurements with Volpara (version 1.5.1) and Quantra (version 2.0), in comparison to visual assessment utilizing the BI-RADS 5th edition [75]. FFDM mammography examinations were retrospectively analyzed. Agreement of density category ranged from moderate to substantial in Quantra, and fair to moderate in Volpara. Assignment of density categories differed significantly between visual and volumetric measurements (*p* < 0.0001); with Quantra assigning lower density categories more frequently than by use of the visual assessment, or Volpara. Conversely, Volpara assigned the extremely dense category more frequently than visual assessment or Quantra. There were statistically significant differences found between Volpara and Quantra when assessing all volumetric data, though they were well correlated (*y* = 0.79–0.99).

While more repeatable than visual assessments, user-assisted methods are dependent on the experience and training of the reader. A study looking at the inter-reader and intra-reader variability using Cumulus software found higher inter-reader agreements for clinically-trained (i.e., radiologists) versus non-clinically trained (i.e., physicists) readers [76]. Fully automated methods, in contrast, may be more repeatable, giving the same measurement on a given image. A comparison of Cumulus, CumulusV, Volpara and Quantra on MBD measurements was conducted at the University of Virginia School of Medicine. Density measurements were obtained for women undergoing same-day repeat mammograms (FFDM) and demonstrated that Volpara and Quantra had the highest reliability [77].

### 6.3. Image Post-Processing Effects

The advent of FFDM provided numerous possibilities for quantitative MBD assessment that were not feasible with film screen mammography. However, variability in MBD assessment can be introduced by the different manufacturer post-processing algorithms applied to digital images to enhance their appearance for radiologist interpretation [78]. Since semi- and fully-automated methods rely on pixel intensity values in the image, any alterations in the relative pixel intensity values within a given image can affect MBD assessments [79]. The concern around a lack of a standardized method for the generation of presentation images is that the MBD assessments may not be consistent, especially when comparing MBD across populations imaged on different manufacturers’ X-ray systems. At least two studies have suggested that visual density assessments may be higher, for example, on GE (General Electric, Waukesha, WI, USA) versus Hologic images [80,81]. Some methods avoid this issue by assessing MBD from the raw mammographic image, but one drawback for these methods is that retrospective studies can be more difficult due to the lack of availability of stored raw images.

Another source of variation in MBD assessment is the synthetic 2D mammogram, which is constructed from multiple projections of digital breast tomosynthesis [82]. C-View (Hologic Inc.), Insight 2D (Siemens AG, Erlangen, Germany) and V-Preview (GE) were developed as an alternative for FFDM during acquisition of tomosynthesis studies with the goal of reducing dose to the patient by doing away with also requiring a set of conventional (2D) mammograms [83]. Currently, these three technologies have been cleared for mammographic screening in the US (with varying indications). It is not clear whether synthetic mammograms can be reliably used for visual density assessment; one previous study has showed shifts within BI-RADS categories 2–4 when C-View was used in place of FFDM [84]. The research team from University of Pennsylvania that developed LIBRA software published results from their evaluation of the agreement between automated estimate of breast density from standard and synthesized mammograms [85]. Briefly, the LIBRA software generates area-based measurements of breast area, dense tissue area, and percentage density from FFDM images. For the purposes of this investigation, the LIBRA algorithm was extended to be able to generate breast density estimates from synthetic images. Results were promising, as the synthesized 2D was found to perform comparably to automated estimates of MBD from the processed 2D mammogram; however, comparisons of MBD assessments on synthesized 2D views from different manufacturers are currently lacking. Volumetric methods have also shown promising results in assessing MBD from both conventional FFDM and DBT data [86,87]. In the study by Pertuz et al. [86], conducted at University of Pennsylvania, correlations of 0.84 and 0.83 were reported comparing Volpara’s estimate of MBD on FFDM with MBD assessed from MRI and DBT reconstructions, respectively. This is important as DBT moves towards becoming the new standard of care for breast screening.

## 7. The Current Clinical Landscape of MBD

### 7.1. MBD and Mammographic Sensitivity

Increased MBD affects various aspects of mammography screening performance. One driver of this is the aforementioned reason of both dense tissue and tumors attenuating X-rays in a similar manner. This contributes to higher rates of interval cancers (cancers that are detected, often symptomatically, between regular screening rounds) in women with higher MBD [21]. Such interval cancers are counted as false negatives during mammography and lead to decreased sensitivity of mammography screening programs. Aside from the masking risk, interval cancers can also be attributed to overlooked cancer features due to the subtlety of presentation, incorrect interpretation of visible signs, or lack of visualization on mammography views due to anatomic location.

Results from large scale studies and screening populations have suggested that for women in the highest density categories, up to 50% of cancers are not detected by mammography (Table 2) and are approximately 6-fold more likely of being diagnosed with an interval cancer compared to those in the lowest two BI-RADS categories [9]. As demonstrated by studies by Pisano and Prummel, sensitivity does differ between film-screen and FFDM [88,89]. Furthermore, women in the highest SCC category have approximately 17-fold risk of being diagnosed with an interval breast cancer compared to women in the lowest SCC category [6]. A European study by Wanders et al. found that Volpara is also associated with increased interval cancer rates for women, with interval cancer rates increasing from 0.7% to 4.4% across VDG categories 1 to 4, respectively [90]. Research we carried out at our center highlighted some of the limitations of using categories of MBD for the assessment of mammographic sensitivity [5]. Continuous VBD measurements allow for a finer discrimination of the mammographic sensitivity for women within a given BI-RADS or VDG category.

Other methods of evaluating the masking risk aspect of mammographic sensitivity have been investigated. An automated, quantitative algorithm was developed that estimates the likelihood of masking of simulated masses by dense tissue [91]. Holland et al. investigated three metrics (percent dense volume, percent dense are where tissue thickness exceeds 1 cm, and dense tissue masking model) for their ability to identify women at high risk for a masked tumor, by evaluating 111 women with interval cancer, and 1110 normal screenings without cancer from the Dutch breast cancer screening program [92].

### 7.2. US Density Notification Legislation

Even though breast density has been acknowledged in the medical community since the 1970s, it has not been widely applied to medical practice until the 2000s. While radiologists have long acknowledged the reduced sensitivity of mammography for women with increased MBD, this information was not, until recently, passed onto the women themselves. Because of being told her cancer may have been missed as a result of high MBD, a grassroots campaign called “Are You Dense?” was initiated, which aimed to spread information to the public about the risks and challenges associated with increased MBD [93]. In 2009 this led to Connecticut becoming the first US state to pass legislation mandating that women with dense breasts (BI-RADS c or d) be informed of the fact and that supplemental screening may be beneficial [94]. As of May 2017, 31 states have some form of density notification law; while some states have efforts for breast density reporting/education, but do not require notification. An additional 10 states have an active bill pending regarding notification [95]. There is no standard from state-to-state on what is told to patients and how they are informed. A federal bill currently introduced to the US Congress would require mammography facilities to report breast density information to physicians and patients [96].

Twenty-four of the states require the use of specific language. Twenty-one states notify women with dense breasts; ten of the states choose to notify everyone, while Oregon only notifies women with “extreme density” (BI-RADS d). Most states (27) inform a woman if she has dense breasts, although in a majority of cases her personal density category is not specified. Twenty-four states mention the masking effect of density and a majority specify it is a risk factor for breast cancer. Unfortunately, less than half (15) mention supplemental screening. Of the 31 states with notification legislation, only 5 states (CT, IL, NJ, NY, IN) have enacted legislation mandating some form of insurance coverage for supplemental screening for women with dense breasts. In cases where “dense breasts” has been defined in the legislation, it is the BI-RADS density categories that have been cited. Therefore, to comply, visual BI-RADS or commercial software that provides density categories must be used i.e., Volpara, Quantra, Philips Spectral Density or iReveal. Providing accurate MBD assessments for communicating masking risk to lay women is becoming increasingly important as more and more states implement density notification laws and the use of objective methods have been shown to improve consistency across radiologist visual readings [97].

### 7.3. Supplemental Screening

Identifying women with dense breasts is paramount for providing them optimal screening outcomes. As it has been established that these women suffer from poor sensitivity on mammographic screening (Table 2), they are excellent candidates for supplemental screening technologies. ACRIN 6666 evaluated an elevated-risk population, which was enriched with dense breasts, and reported a sensitivity of mammography of 50%; mammography plus ultrasound increased sensitivity to 77.5% [98]. The follow-up study supported initial findings, and support that it may be reasonable to offer supplemental screening ultrasound to women with dense breasts, in both the high risk and intermediate risk categories [99]. Automated breast ultrasound is also being investigated in the setting of evaluating women with dense breast tissue. The Invenia automated breast ultrasound system (ABUS) is the only FDA-approved automated breast ultrasound for screening women with dense breast tissue [100]. A recent 2016 publication [101] conducted in Sweden evaluated the impact of ABUS when added to FFDM on breast cancer detection and recall rates in a group of asymptomatic women with dense breasts. Combined, FFDM and ABUS had a cancer detection rate of 6.6 cancers per 1000, compared with 4.2 per 1000 with FFDM alone. Recall rate did increase for combined FFDM and ABUS (22.8 vs. 13.8, respectively). Large-scale studies from the US [102] and Europe [103] have shown that DBT provides improved screening performance for women with dense breasts compared to FFDM [104]. Similar findings are noted for ultrasound [105] and MRI [106]. Molecular breast imaging (MBI) has also proven to be advantageous for women with dense breasts [107], though research is limited regarding this. The Dense Tissue and Early Breast Neoplasm Screening (DENSE) randomized trial currently underway within the Dutch breast screening program is a large-scale study to investigate supplemental MRI in women with dense breasts [108]. Results will prove informative regarding whether supplemental MRI can decrease the rate of interval cancer in these women and whether such a screening modality is cost-effective [109].

Currently in the US, even in states with density notification legislation in place, uptake of supplemental screening is fairly low. A previous study in our clinic found a 2% uptake of supplemental screening ultrasound for women notified of their dense breast tissue in an initial period after implementation of the law in our state (NY) [110]. Further review of our patient population has showed a steady increase in adoption. The wording of the notification letters is an important consideration, as some (like the NY legislation) suggest further discussions with primary care physicians, which may reduce the numbers of women scheduling same-day screening ultrasounds. What impact the recent NY legislation mandating insurance coverage will have on uptake of supplemental screening is yet to be seen.

One hindrance to more widespread use of supplemental screening is the large financial cost associated. For example, the 2017 reimbursement (based on Medicare average) for screening bilateral breast ultrasound is $156.98; which does not fully cover the cost a facility incurs to offer this service. Screening bilateral breast MRI reimbursement is approximately $518.68 (based on Medicare), similarly not covering the costs associated with a facility offering the service.

The American College of Radiology (ACR) Appropriateness Criteria [111] warns that screening ultrasound may not be a cost-effective practice due to a high false-positive rate and time-consuming nature of the exam (handheld). An analysis of the cost effectiveness of screening ultrasound for women with dense breasts by Sprague et al. [112] measured breast cancer deaths averted, quality-adjusted life years gained (QALY), false positives, costs, and costs per QALY gained. When reviewing the age group of 50–74, supplemental screening ultrasound averted 0.36 additional breast cancer deaths; gained 1.7 QALYs, and resulted in 354 false-positive biopsy recommendations. Cost-effectiveness ratio was $325,000 per QALY gained; when looking at only those with extremely dense breasts, the cost was $246,000 per QALY gained. The study findings seem to indicate that supplemental ultrasound in women with dense breasts would substantially increase costs, with small benefits in QALYs and deaths averted.

A review of the literature shows that supplemental screening does, in general, consistently detect additional breast cancers, most of which are invasive [106], though many of these supplemental tests lead to additional recalls and biopsies. However also of note is that there are not many published studies evaluating supplemental screening modalities specifically in women with dense breasts.

The cost-effectiveness of screening breast MRI is limited, and has largely been evaluated in high risk populations. A notable study [113] evaluated cost-effectiveness for adding MRI to mammography screening for women with a *BRCA1* or *BRCA2* mutation. The QALY saved varied by age, and was more favorable to those with a *BRCA1* mutation. Interestingly, the study did find that cost-effectiveness increased when mammography sensitivity was lower, particularly in women with very dense tissue. According to the ACR Appropriateness Criteria, screening those at high risk with MRI is cost-effective, and this increases with increasing breast cancer risk. However, women with dense breast tissue are not considered in this.

## 8. MBD and Breast Cancer Risk

A link between MBD and breast cancer risk was first proposed in 1976 by the radiologist John Wolfe; however, several studies [21,22,23,24,25,26] were unable to reproduce Wolfe’s findings and his hypothesis fell out of favor for a number of years, only to re-emerge in the 1980s. Several studies have now definitively established MBD as being an independent risk factor for breast cancer [6,114,115]. The highest categories of MBD are reported to confer relative risks (RR) of 4–8-fold compared to the lowest MBD categories, or approximately 2-fold compared to the population average breast density. For comparison, the RR conferred by having a first degree relative with breast cancer is approximately 2-fold [116]. It is estimated that approximately 43% of screening aged women in the US have dense breasts [117], and due to the prevalence of increased MBD in the population, MBD is thought to account for 16–30% of breast cancers [6,19,118]. A recent study estimated that 26–39% of breast cancers could be prevented if women shifted from dense to non-dense categories [119]. Furthermore, extended follow-up indicates that density remains associated with risk for between 4 to 8 years after study entry and density assessment: OR (odds ratio) 3.7 (95% CI (confidence intervals) 1.5–93) for screen-detected cancers, OR 8.9 (95% CI 2.8–28.6) for cancers detected by other means. Overall cancer risk remained significantly elevated (OR 4.47; CI 95% 2.1–9.6) for a decade or more since the initial density assessment (when comparing visually assessed density of ≥75% to 0% density) [117].

It should be noted that different methods of MBD measurement bear different levels of association with breast cancer (Table 3, Table 4 and Table 5). Several factors must be considered when making comparisons between different studies, as they limit the appropriateness of such comparisons. Such factors include the study population (and the population-specific distribution of density and disease prevalence), the reference category used, adjustments for covariates made during analysis, and the image type, to name a few. It should also be considered that the risk association of any one density method varies between studies; there is often an overlap between the risk associations of different methods. Nevertheless, when considering the maximum risk association of any of the reported studies, the qualitative BI-RADS reached a maximum OR of 4.08 (95% CI 2.96, 5.63) across nine studies considered in this review. Meanwhile, the semi-quantitative SCC method reached a RR of 6.05 (95% CI 2.82, 12.97) when women with ≥75% density were compared to those with 0% density. However, the number of women in the reference groups of the two methods is likely to be different; thus, it is not possible to ascertain which of these visual methods bears the strongest association with risk. To allow for more uniform comparison between the various methods, we looked at the risk associations on a quintile basis—where the risk of women in the top 20% of density values is compared to that of women in the lowest 20% of density. The semi-quantitative VAS showed a maximum OR 4.85 (95% CI 3.00–7.83) when considering the risk of future cancer development. The maximum risk association of the quantitative area-based measures tended to be lower; it ranged from OR 2.07 (95% CI 1.12, 3.83) for LIBRA (when using display images) to 3.38 (95% CI 2.0–5.72) for Cumulus. However, the maximum risk association exhibited by quantitative volumetric tended to be higher, ranging from OR 3.94 (95% CI 2.26, 6.86) exhibited by Quantra to OR 8.26 (95% CI 4.28, 15.96) shown by Volpara. Therefore, while there is considerable overlap in the risk associations exhibited by different measurement methods, there are instances where volumetric methods give the strongest association to the disease.

It is difficult to make conclusions about how mammography type (film versus FFDM) affects the association between density and breast cancer risk, and how this differs between measurement methods. Risk associations for many of the more recent methods have only been published for a single mammography type—either because the methods obligately require raw images, or simply due to lack of studies available. BI-RADS and Cumulus are some of the few methods that have more than one study for each mammography type. For these two methods, it is notable that film screen mammography appears to produce higher maximum risk associations than seen on FFDM (Table 3 and Table 4). Both BI-RADS and Cumulus require radiologist input to assess density; thus, it is possible that vendor-specific image processing applied to FFDM images may affect radiologists’ judgements of density, which may in turn affect risk associations [120]. However, it should be noted that there is considerable overlap in the ORs derived from film and FFDM images. Furthermore, a study that quantified the risk association of Cumulus on FFDM compared to “analogue-like” images found that FFDM produced the higher OR [121].

A number of studies have applied multiple MBD measurement methods on the same cohort of women, allowing for direct comparisons to be made. Some studies have concluded that visual assessment methods are most indicative of breast cancer risk. Researchers involved in the Predicting Risk of Cancer at Screening (PROCAS) study have found VAS to have a greater association with cancer risk, both for screen-detected as well as future cancers [34,122]. Similarly, researchers at the Mayo clinic have found BI-RADS to produce a higher OR than volumetric methods [61]. However, it should be considered that with quantitative/semi-quantitative methods, such an association may not be recapitulated with a different set of readers [73,123]. Conversely, several other studies that included both qualitative and quantitative methods of assessment have found quantitative measures to produce a better prediction of risk [44,114,124]. A meta-analysis of breast cancer incidence studies from the general population has shown that the top density category confers increased RR of cancer compared to the least dense category: RR 3.98 for qualitative density (Wolfe DY vs. N1); RR 4.64 for quantitative density (visually estimated density of ≥75% vs. <5%) [125]. Finally, three studies have compared area-based and volumetric measures, with volumetric measures showing a greater association with breast cancer [61,121,126].

### 8.1. Incorporation of MBD into Risk Prediction Models

Despite being one of the strongest risk factors for breast cancer, MBD is not routinely used for breast cancer risk assessment. Incorporation of BI-RADS categories or an area-based continuous measure of MBD into current risk prediction models, such as the Gail and Tyrer-Cuzick models, have only showed minimal to modest improvements in terms of discriminatory ability [129,146,147]. Formal risk assessment of breast cancer (through family history or with mathematical risk models that are “capable of pedigree analysis of first-degree and second-degree relatives on both the maternal and paternal sides”) allows at-risk women to be considered for disease-preventative measures (such as preventative therapies, genetic testing or MRI screening) [148,149,150]. Currently, only two risk models that include MBD are freely available to the public—the Breast Cancer Surveillance Consortium (BCSC) model and the Tyrer-Cuzick model. The BCSC model use BI-RADS density categories as the density input, whereas the Tyrer-Cuzick version 8 [151] allows density inputs, from an automated density assessment, VAS or BI-RADS categories. As Tyrer-Cuzick is well accepted by advisory bodies such as the American Cancer Society, the new incorporation of breast density will mean that this important risk factor is taken into consideration for official recommendations on supplementary screening and risk minimization strategies. As a large proportion of women fall into the middle two BI-RADS categories, the use of the continuous measures of MBD can allow for better risk discrimination. Furthermore, as discussed above, reader-dependent measures of MBD may be limited (VAS and BI-RADS), because of their subjectivity. Finally, volumetric and area-based methods quantify MBD differently and thus are not equivalent inputs for a risk model. In addition to the points discussed in the section “Area vs. volumetric MBD”, volumetric methods have recently been shown to be effective in tracking MBD reduction following intervention by three different estrogen receptor modulators [152]. The cited systematic review has indicated that area-based methods do not consistently show a reduction in MBD in response to the same agents. While more study is required, this may suggest that volumetric methods may better show changes in breast density following interventions on breast density, and therefore breast cancer risk.

### 8.2. MBD and Breast Cancer Prognosis

Not only does dense tissue influence the risk of developing cancer, some studies have found increased density is related to poorer prognostic features, recurrence and survival, though results are variable. One study found a higher risk of subsequent breast cancer among patients with ductal carcinoma in situ (DCIS) with highly dense breasts [153], and higher local recurrence rates with higher density has also been reported [12,154]. Reports on mammographic density and breast cancer survival are mixed; breast density was found to be significantly associated with breast cancer incidence and breast cancer mortality in a Swedish population [155], while two other studies did not find an adverse effect on survival [156,157].

Conversely, interval breast cancers in non-dense breasts were associated with lymph node involvement (OR 3.55), as well as estrogen receptor negative status (OR 4.05), human epidermal growth factor receptor 2 positive (OR 5.17), progesterone receptor negative (OR 2.63), triple negative (OR 5.33), grade 3 disease (OR 3.43), and tumor size >40 mm (OR 4.90) [158]. In comparison, interval cancers in dense breasts were less aggressive, and were phenotypically similar to screen-detected cancers. When comparing interval to screen-detected cancers, high mammographic density was more common in patients with interval cancers, as reported by other studies.

Breast cancer specific survival, when taking mammographic density into consideration, was explored comparing interval cancers and screen-detected cancers [13]. Utilizing Cumulus to assess density, the study showed that women with interval cancers in nondense and dense breasts had poorer survival than those with corresponding screen-detected cancers. Researchers reported that potentially the claim could be made that poorer prognosis in women with dense breasts and an interval cancer could be due to later detection of the tumor, possibly demonstrating the need for higher sensitivity in screening technologies, though more work would be needed to support this.

Mammographic density was not found to be associated with breast cancer-specific survival (hazard ratio, (HR) 0.95) in a review of 607 breast cancer cases [159]. However, the interaction with radiotherapy was highly significant (*p* = 0.0006). Percent density was associated with reduced risk of dying from the disease in those who received radiation, but with an elevated risk in those who did not (HR 0.77 vs. HR 1.46, respectively). This work suggests additional value of assessing breast density, as it may aid in identifying women with a poorer prognosis and allow for recommendation of radiotherapy to improve outcomes. Similarly, review of data from the US BCSC did not find an association between high mammographic density and risk of death from breast cancer [157]. An important conclusion drawn from this is that perhaps risk factors for developing breast cancer are not the same as those influencing the risk of death from the disease.

While density is not currently considered in determining breast cancer prognosis, the improvement in the accuracy and reliability of MBD assessment methods could see MBD incorporated into prognostic determinations in the future.

### 8.3. Longitudinal Changes in MBD

MBD does not remain steady during a woman’s lifetime. Breasts undergo age-related involution which has an inverse association with density [160]. Menopause, in particular, is associated with a 2.4% drop in percent area mammographic density [161]. Initial breast density at the start of a measurement period also affects overall density change; women with high density undergo a greater total decline of density with age compared to those with lower baseline density [162]. Extrinsic hormones and medication impacts density in several ways. Hormone replacement therapy (HRT) used to alleviate menopausal symptoms (particularly combination HRT that uses estrogen and progesterone) leads to increased density [163,164,165]. A recent study from the Women’s Health Initiative (WHI) has shown that combination HRT is associated with increased breast cancer risk, and that increased risk is mediated almost entirely by increased breast density [166]. Conversely, tamoxifen (a selective estrogen receptor modulator, SERM, which blocks the activity of estrogen inside cells) leads to density decreases for some [132,167]. Because of all these factors, multiple studies have documented the changes in density over time [168,169,170,171,172]. Changes in density mean that a woman is not likely to remain at the same level of risk throughout her life, in terms of masking mammographic sensitivity and de novo cancer development. Thus, accurate longitudinal measurement of MBD is important for optimizing a woman’s health care.

When MBD assessments are made in normal clinical practice, the density scores of the prior exams are generally available. This could influence the final density score, as it is more likely that changes in density scores will only occur if significant changes are observed visually. Objective and automated methods (particularly, volumetric methods, as discussed above) may be able to better show changes in breast density following interventions, and thus may be more appropriate for monitoring the efficacy of such interventions in reducing risk through breast density. Furthermore, depending on the method, small changes in density may go undetected. For instance, Cuzick et al. noted that a 10% change in density as measured by VAS was the smallest change that could be detected reproducibly [132]. This is another area where objective (particularly continuous) methods may offer an advantage.

A recent study compared BI-RADS to an automated volumetric density measure in the Dutch breast screening program to determine which is more appropriate for temporal measurements [92]. Five hundred women were randomly selected from the program; each had a “prior” and a “current” mammogram, with an average 30-month interval between them. Density was established (either by BI-RADS 4th edition readings or by the BI-RADS-like categories provided by the automated software) on a two-category (“fatty” versus “dense”) or a four-category (BI-RADS 1, 2, 3 or 4) scale. The automated software produced a significantly higher portion of women who did not exhibit a change between two-point density categories (90.4% of women) compared to the group reading of radiologists (86.8%). This may reflect the fact that the software produced more consistent density readings than radiologists did—an idea supported by the fact that the software’s agreement to its own readings between serial exams was significantly higher than the group radiologist readings were to each other. On a two-category scale, the software maintained a kappa agreement value of 0.8 across screening exams, while the group radiologist readings had a kappa of 0.7; on a four-category scale the κ values were 0.85 and 0.75 respectively. When women did exhibit a density change between screens, most of the instances of change were from the “dense” to “fatty” category (this happened in approximately 70% of cases of density change)—as would be expected for age-related involution or menopause transition. Thus, an objective measure may be preferable to produce more accurate temporal density readings.

### 8.4. Reducing Breast Density: Reducing Risk?

Tamoxifen treatment reduces breast density; reportedly in 30–60% of breast cancer cases [173,174]. An ongoing investigation into this is being conducted in Sweden [175]. The study’s primary aim is to identify the minimum dose of tamoxifen non-inferior in its ability to reduce mammographic density and with fewer side effects compared to 20 mg of tamoxifen. Association with reduced risk of recurrence and mortality in breast cancer patients, as well as reduced risk of breast cancer in those utilizing the drug for preventative reasons has been noted, with reductions in breast density from tamoxifen reportedly 10–20% [132,167,176,177,178]. Tamoxifen has been investigated in patients with dense breast tissue due to the known benefits, but it was not well studied if tamoxifen-induced breast density reductions could identify women who would benefit from prophylactic treatment with the drug [132]. Cuzick et al. reported 46% of women treated with tamoxifen had a 10% or greater reduction in MBD by 12- to 18-month mammogram [132]. These women, when compared to those in the placebo arm, had a 63% reduction in breast cancer risk (OR 0.37; 95% CI 0.20, 0.69), while women who received tamoxifen but did not achieve the 10% reduction in MBD underwent no significant decrease in risk, relative to the placebo arm (OR 1.13; 95% CI 0.72, 1.77). It should be noted that it is not possible to establish from these results that the observed risk reduction is mediated entirely through the tamoxifen-mediated MBD reduction; however, these results suggest that change in mammographic breast density can be a predictor of response to tamoxifen when used in the preventive setting. This is of clinical utility, as tamoxifen requires prolonged administration and is associated with a range of adverse effects [179,180]. However, tamoxifen needs to be converted to its active metabolite forms (the chief of which are 4-hydroxytamoxifen and endoxifen) by the P450 2D6 metabolic enzyme (encoded by *CYP2D6*) before it can take effect [181]. The considerable prevalence of polymorphisms in *CYP2D6* results in some women being poor tamoxifen metabolizers, and failing to attain clinical benefit due to a lack of active drug forms. Thus, a biomarker such as MBD reduction is valuable in order to identify women who are likely to benefit from treatment.

More recently, reductions in breast density with tamoxifen and aromatase inhibitors (AI) as a marker of treatment response was investigated in women with breast cancer, by comparing to a control group of untreated women without breast cancer; the first study of its kind to validate automated measures of breast density [182]. Declines in volumetric percent density were noted in patients treated with both tamoxifen and AI; greatest reductions in women with ≥10% baseline density. The study confirmed that automated software can detect volumetric breast density changes in women treated with both tamoxifen and AI; suggesting that if these volumetric density declines can predict breast cancer outcomes these measures could be used as prognostic indicators.

Change in density has been discussed as a biomarker for assessing risk [183]. Breast density as a prognostic marker of response to adjuvant tamoxifen therapy has been investigated [167]. In a cohort of postmenopausal breast cancer patients, women treated with tamoxifen and experienced a relative reduction in density of more than 20% between baseline examination and first follow-up mammogram had a 50% reduced risk of death from breast cancer when compared to those with stable density. There was no statistically significant association between density change and survival in those who did not take tamoxifen. This suggests that decrease in density after breast cancer diagnosis can be a prognostic marker for improved long-term survival in patients treated with adjuvant tamoxifen.

It should be noted that both tamoxifen and aromatase inhibitors act through the estrogenic pathway to inhibit cell proliferation [184]. Thus, the above-mentioned discussion of breast density as a biomarker of breast cancer risk reduction is likely only applicable to cases of ER-positive breast cancers. In addition to its estrogenic effects, tamoxifen reduces signaling through the insulin-like growth factor (IGF) pathway and reduces levels of IGF-I in circulation [185,186]. Signaling through the IGF pathway stimulates cell proliferation and has been linked to both increased cancer risk and increased MBD in pre-menopausal women [187,188]. However, breast density and cancer risk are also affected by a milieu of other cellular factors, such as collagen content, extra-cellular matrix (ECM) stiffness and inflammatory factors, among others [189,190]. Thus, the relationship between chemopreventative measures, MBD and breast cancer risk is very complex, with multiple contributing factors.

A recent large-scale study evaluated population-attributable risk proportion for breast cancer associated with clinical breast cancer risk factors in premenopausal and postmenopausal women [119]. Over 50% of breast cancers in each group could be linked to commonly collected risk factors, and researchers state that a substantial proportion of breast cancer can be attributed to high breast density alone, leading to suggestion that behaviors or interventions that reduce breast density could potentially eliminate a large proportion of breast cancers in both pre- and postmenopausal women. The study results suggest that a shift down in breast density of a single category would result in a substantial reduction in breast cancers in the population, a finding that has been also reported previously [8,191] and propose means of doing so could be increased breastfeeding, or prevention with tamoxifen. The authors caution that these interventions may effectively reduce breast density, but should be considered carefully in context with potential harms.

## 9. Conclusions

MBD has come to be well-established as an important risk factor for breast cancer and an important consideration for breast cancer screening. Reliable assessment is also important for temporal assessment of breast density, in order to accurately characterize a woman’s breast cancer risk throughout her lifetime. Having clinically proven methods for breast density assessment are essential for providing women with optimal health care. The earlier qualitative measurement methods have limited consistency between readers and in relation to breast cancer risk. However, certain studies have demonstrated that the visual assessment of MBD may be detecting aspects of fibroglandular tissue that the computer-based methods do not. The development of automated computer-based density methods are advantageous in that they have been shown to provide consistent, reproducible and objective results, and can be implemented in large-scale clinical settings, such as breast screening programs. Moving towards a standardized assessment of MBD for clinical applications, while desirable, is a hugely complex feat. One must consider not only whether one method is better able to predict breast cancer risk or mammographic performance, but how consistent the method is across X-ray system vendors, modalities and over time, as well as how feasible the method is in terms of integration into health information technology (IT) systems and clinical practice. Although the MBD landscape has evolved rapidly since its inception in 1976, there is currently no consensus as to which methods are most appropriate for tailoring interventions to improve the early detection of breast cancer or reduce breast cancer risk. As demonstrated by the increasing interest in the development of MBD assessment methods, this is a highly active area of research. This research activity is expected to provide continuing improvement in MBD measurement, which will in turn translate to better risk assessment for women, high quality decision making when offering supplemental screening and improved monitoring of density over time.

## Figures and Tables

**Table 1:** BI-RADS 4th and 5th edition category definitions.

BI-RADS 4th Edition		BI-RADS 5th Edition	
1	The breast is almost entirely fat (<25% glandular)	a	The breasts are almost entirely fatty
2	There are scattered densities (approximately 25–50% glandular)	b	There are scattered areas of fibroglandular density
3	The breast tissue is heterogeneously dense, which could obscure detection of small masses (approximately 51–75% glandular)	c	The breasts are heterogeneously dense, which may obscure detection of small masses
4	The breast tissue is extremely dense. This may lower the sensitivity of mammography (>75% glandular)	d	The breasts are extremely dense, which lowers the sensitivity of mammography

**Table 2:** Sensitivity of mammography by density category in US population-based studies.

Study	Population	Period	Number of Women	MBD Classification	Sensitivity		SF vs. FFDM
Mandelson et al., 2000 [9]	US Breast Cancer Screening Program	1988–1993	149 women with interval cancer; 388 women with screen-detected	BI-RADS 3rd ed.	BI-RADS 1 + 2	80.3%	SF
					BI-RADS 3	58.8%	
					BI-RADS 4	30.4%	
Kolb, Lichy, and Newhouse, 2002 [11]	US DMIST Trial	1995–2000	27,825 screening sessions; 246 cancer diagnoses in 221 women	BI-RADS 3rd ed.	BI-RADS1	98%	SF
					BI-RADS 2	82.9%	
					BI-RADS 3	64.4%	
					BI-RADS 4	47.8%	
Carney et al., 2003 [10]	US Breast Cancer Surveillance Consortium	1996–1999	329,495 women; 2223 breast cancer diagnoses	BI-RADS 3rd ed.	BI-RADS1	88.2%	SF
					BI-RADS 2	82.1%	
					BI-RADS 3	68.9%	
					BI-RADS 4	62.2%	
Boyd et al., 2007 [6]	National Breast Screening study (Canada)	1981–1990	45,000 women	SCC	<10%	75.2%	SF
					10–25%	62.9%	
	Screening Mammography Program of British Colombia	1993–1999	254,082 women		25–50%	65.2%	
					50–75%	57.3%	
	Ontario Breast Screening Program	1996–2003	166,254 women		≥75%	54.2%	
Kerlikowske et al., 2007 [8]	US Breast Cancer Surveillance Consortium	1996–2003	1,714,351 women	BI-RADS	BI-RADS1	89%	SF
					BI-RADS 2	84%	
					BI-RADS 3	77%	
					BI-RADS 4	64%	

Abbreviations used: FFDM, full field digital mammography; SF, screen film.

**Table 3:** Association of breast cancer risk with visual methods of measuring MBD.

Method				Risk Association	Reference Group	Adjustment	Population (*n*)	Country	Postmenopausal %	Image Type	Reference
Visual	Area-based	Parenchymal patterns	Wolfe patterns	RR 3.98 (95% CI 2.54, 3.66) incidence studies; RR 2.42 (95% CI 1.98, 2.97) prevalence studies	DY vs. N1	Meta-analysis				Film	McCormack 2006 [125]
			Tabar	2.42-fold risk increase	Pattern IV vs. pattern I (pattern V—no increase)	None	174	Singapore	89%	Film	Jakes 2000 [127]
		Qualitative	BI-RADS^®^	HR 2.09 (95% CI 1.59, 2.75)	BI-RADS 4 vs. 2 (3rd ed.)	A, BMI, FH, HRT, M, P, R	44,811	USA	58.1% post- or perimenopausal	Film	Ziv 2004 [128]
				OR 3.93 (95% CI 2.46, 6.28) premenopausal; OR 3.15 (95% 2.72, 3.66) postmenopausal	BI-RADS 4 vs.1 (4th ed.)	A, FH (1st degree), MBD, prior breast procedure; if postmenopausal, also—BMI, FB, Hispanic ethnicity, HRT, previous mammographic outcome, R, surgical menopause	1,007,600	USA	74.3%	Film	Barlow 2006 [129]
				RR 4.08 (95% CI 2.96, 5.63)	BI-RADS 4 vs. 1 (3rd ed.)	Meta-analysis				Film	McCormack 2006 [125]
				Incidence rate ratio 2.45 (95% CI 2.14, 2.81)	BI-RADS 3 and 4 vs. 1 and 2 (4th ed.)	A	48,052	Denmark	Not reported	Film	Olsen 2009 [130]
				OR 2.96 (95% CI 0.50, 17.49)	BI-RADS 4 vs. 1 (4th ed.)	A, BMI, M, P	1099	UK	86.4%	FFDM	Eng 2014 [114]
				OR 1.19 (95% CI 0.33, 4.33)	BI-RADS 4 vs. 1 (4th ed.)	A, BMI, FH, Men, PrevBiop, R	424	USA	Not reported	FFDM	Keller 2015 [44]
				OR 2.29 (95% CI 1.87, 2.81)	BI-RADS 4 vs. 2 (4th ed.)	A, BMI	6081	USA	Both, breakdown not reported	FFDM	Brandt 2016 [61]
				OR 2.03 (95% CI 0.85, 4.97)	BI-RADS D vs. B (ed. not reported)	BMI, M, P	399	USA	67.2%	FFDM	Jeffers 2016 [124]
				OR 1.81 (95% CI 1.65–1.99) premenopausal; OR 1.58 (95% CI 1.46, 1.71) postmenopausal	BI-RADS D vs. B (ed. not reported)	BMI, FB, FH, history of benign breast biopsy	202,746	USA	71.3%	Not reported	Engmann 2017 [119]
		Semi-quantitative	Boyd categories	RR 6.05 (95% CI 2.82, 12.97)	≥75% vs. 0% density	FB, FH, height, Men, P, weight	310	Canada	Not reported	Film	Boyd 1995 [29]
				OR 4.7 (95% CI 3.0, 7.4)	≥75% vs. <10% density	A, age at menopause, BMI, FB, FH (1st degree), HRT M, Men, observation time P, study	2224	Canada	75.4%	Not reported	Boyd 2007 [6]
				OR 3.5 (95% CI 2.0, 6.2) screen-detected cancers only	≥75% vs. <10% density	A, age at menopause, BMI, FB, FH (1st degree), HRT M, Men, observation time P, study	1434	Canada	75.4%	Not reported	Boyd 2007 [6]
				OR 3.55 (95% CI 0.78, 16.09)	≥75% vs. ≤5% (MODIFIED SCC)	A, FH (1st degree), HRT M, P	1287	Canada	75.3%	FFDM	Abdolell 2014 [131]
			Visual analogue scale	OR 3.43 (95% CI 1.43, 8.19)	76–100% vs. 0%	A, atypical hyperplasia or LCIS, BMI, HRT	1065	UK, Finland	46.5%	Film	Cuzick 2011 [132]
				OR 1.48 (95% CI 1.34, 1.63)	Density residual 75th vs. 25th percentile	A, BMI, mammography type	50,628	UK	72%	~20% film, remainder FFDM	Brentnall 2015 [133]
				OR 4.64 (95% CI 2.84–7.56); screen-detected cancers	Quintile 5 vs. 1	None specified	1464	UK	Not reported	FFDM	Astley 2016 [122]
				OR 4.85 (95% CI 3.00–7.83); future development of cancer	Quintile 5 vs. 1	None specified	1352	UK	Not reported	FFDM	Astley 2016 [122]
				OR 2.12 (95% CI 1.59, 2.84) univariate analysis; OR 2.75 (95% CI 1.99, 3.81) multivariate analysis; screen-detected cancers	Quartile 4 vs. 1	None	1296	UK	Not reported	FFDM	Evans 2016 [34]
				OR 3.59 (95% CI 2.37, 5.43); future development of cancer	Quartile 4 vs. 1	When adjusted: A, BMI, M	33,142	UK	Not reported	FFDM	Evans 2016 [34]

Abbreviations: A, age; BMI, body mass index; CI, confidence intervals; FB, age at first birth; FH, family history of breast cancer; HR, hazard ratio; HRT, use of hormone replacement therapy; M, menopausal status; Men, age at menarche; OC, oral contraceptive use; OR, odds ratio; P, parity; PrevBiop, number of previous biopsies; R, race; RR, relative risk.

**Table 4:** Association of breast cancer risk with area-based methods of measuring MBD.

Method				Risk Association	Reference Group	Adjustment	Population (*n*)	Country	Postmenopausal %	Image Type	Reference
Semi-automated	Area-based	Quantitative	Cumulus	RR 4.04 (95% CI 2.12, 7.69)	>75% vs. 0% density	FB, FH, height, Men, P, weight	310	Canada	Not reported	Film	Boyd 1995 [29]
				OR 5.86 (95% CI 2.2, 15.6)	>75% vs. 0% density	A, age at menopause, BMI, FB, FH, HRT, M, Men, observation time, P, study	2228	Canada	65.3%	Film	Boyd 2006 [134]
				OR 2.19 (95% CI 1.28, 3.72)	Quintile 5 vs. 1	Age, BMI, FB, FH, HRT, M, Men, P	1028	Canada	69.8%	Film	Aitken 2010 [135]
				OR 1.8 (95% CI 1.0, 2.9)	Quintile 5 vs. 1	A, age menopause, BMI, FB, FH, height, HRT, Men, OC, P	1217	Netherlands	100%	Film	Lokate 2011 [136]
				OR 2.72 (95% CI 1.93, 3.83) premenopausal	Tertile 3 vs. 1	A, age at menopause (if postmenopausal), alcohol use, BMI, FB, FH, Men, P, study	4084	USA	64.2%	Film	Pettersen 2011 [137]
				OR 3.28 (95% CI 2.41, 4.45) postmenopausal	Quintile 5 vs. 1						
				OR 2.5 (95% CI 1.5, 4.3)	Quintile 5 vs. 1	A, BMI, FB, FH, history of benign breast biopsy, mammography system, R	1100	USA	67.2%	Film	Shepherd 2011 [126]
				OR 2.47	>25% vs. 0–5% density	None specified	1512	Sweden	100%	Film	Li 2012 [43]
				OR 2.4 (95% CI 1.9, 3.1)	Decile 10 vs. quintile 1 (dense AREA)	A, FH, HRT, screening round, symptoms	6327	Australia	Not reported	Film	Nickson 2013 [138]
				OR 3.38 (95% CI 2.00, 5.72)	Quintile 5 vs. 1	A, BMI, M, P	1099	UK	86.4%	FFDM	Eng 2014 [114]
				OR 1.58 (95% CI 1.33, 1.88)	per SD						
				OR 1.98 (95% CI 1.14, 3.44) raw images; OR 2.90 (95% CI 1.66, 5.06) processed images; OR 3.02 (95% CI 1.77, 5.16) analogue-like images	Quintile 5 vs. 1	A, BMI, HRT, M, Men, OC, P	1098	UK	86.3%	FFDM	Busana 2016 [121]
				OR 1.93 (95% CI 1.12, 3.34) univariate analysis; screen-detected cancer	Quartile 4 vs. 1	None	720	UK	Not reported	FFDM	Evans 2016 [34]
				OR 2.00 (95% CI 1.19, 2.19)	Quartile 4 vs. 2	BMI, M, P	399	USA	67.2%	FFDM	Jeffers 2016 [124]
			Madena	OR 5.23 (95% CI 1.40, 16.13)	≥75% vs. <1% density	A, BMI, FB, FH, HRT, M, Men, P	1065	USA	55.5%	Film	Ursin 2003 [139]
				OR 2.12 (95% CI 1.25, 3.62)	Quartile 4 vs. 1	A, BMI, HRT, M, P	937	Germany	78.2%	Both (proportions not specified)	Rauh 2012 [140]
Fully automated		Quantitative	AutoDensity	OR 3.2 (95% CI 2.5, 4.1)	Decile 10 vs. quintile 1 (dense AREA)	A, FH, HRT, screening round, symptoms	6327	Australia	Not reported	Film	Nickson 2013 [138]
			ImageJ	OR 2.37	>25% vs. 0–5% density	None specified	1512	Sweden	100%	Film	Li 2012 [43]
				OR 2.25 (95% CI 1.46, 4.43)	Quintile 5 vs. 1	A, BMI, M, P	1099	UK	86.4%	FFDM	Eng 2014 [114]
				OR 1.45 (95% CI 1.21, 1.74)	per SD						
			Libra	OR 6.68 (95% CI 2.85, 15.58)	90th vs. 10th percentile	A, BMI, FH, Men, PrevBiop, R	424	USA	Not reported	FFDM	Keller 2015 [44]
				OR 2.24 (95% CI 1.56, 3.21)	per SD increase						
				OR 1.3 (95% CI 1.1, 1.5) processed images; OR 1.1 (95% CI 1.0, 1.3) raw images	per SD increase	A, BMI	1662	USA	Not reported	FFDM	Brandt 2016 [141]
				OR 1.94 (95% CI 1.16, 3.22) raw images; OR 2.07 (95% CI 1.12, 3.83) processed images	Quintile 5 vs. 1	A, BMI, HRT, M, Men, OC, P	1098	UK	86.3%	FFDM	Busana 2016 [121]
			STRATUS	HR 1.6 (95% CI 1.4, 1.8)	per SD increase	A, BMI, FH, HRT, M, masses, microcalcifications	2165	Sweden	65%	FFDM	Eriksson 2017 [45]
				HR 4.8 (95% CI 2.6, 8.8)	BI-RADS-like category (4 vs. 1)						

Abbreviations: A, age; BMI, body mass index; CI, confidence intervals; FB, age at first birth; FH, family history of breast cancer; HR, hazard ratio; HRT, use of hormone replacement therapy; M, menopausal status; Men, age at menarche; OC, oral contraceptive use; OR, odds ratio; P, parity; PrevBiop, number of previous biopsies; R, race; RR, relative risk.

**Table 5:** Association of breast cancer risk with volumetric methods of measuring MBD.

Method				Risk Association	Reference Group	Adjustment	Population (*n*)	Country	% Postmenopausal	Image Type	Reference
Fully automated	Volumetric	Quantitative	BD_SXA_	OR 4.1 (95% CI 2.3, 7.2)	Quintile 5 vs. 1	A, BMI, FB, FH, history of benign breast biopsy, mammography system, R	1100	USA	67.2%	Film	Shepherd 2011 [126]
				OR 2.99 (95% CI 1.76, 5.09)	Quintile 5 vs. 1	A, BMI, M, P	1099	UK	86.4%	FFDM	Eng 2014 [114]
				OR 1.37 (95% CI 1.16, 1.63)	per SD increase						
			CumulusV	RR 2.8	Octile 8 vs. 1	None specified	1158	Canada	Not reported	FFDM	Yaffe 2011 [142]
			Quantra	OR 3.94 (95% CI 2.26, 6.86)	Quintile 5 vs. 1	A, BMI, M, P	1099	UK	86.4%	FFDM	Eng 2014 [114]
				OR 1.40 (95% CI 1.19, 1.66)	per SD increase						
				OR 10.88 (95% CI 4.18, 28.21)	90th vs. 10th percentile	A, BMI, FH, Men, PrevBiop, R	424	USA	Not reported	FFDM	Keller 2015 [44]
				OR 2.64 (95% CI 1.79, 3.89)	per SD increase						
				No association; screen-detected cancer	Quintile 5 vs. 1	None specified	1464	UK	Not reported	FFDM	Astley 2016 [122]
				OR 1.52 (95% CI 1.04, 2.23); future cancer	Quintile 5 vs. 1	Breast volume, P	1352	UK	Not reported	FFDM	Astley 2016 [122]
				OR 1.78 (95% CI 1.46, 2.17)	Quintile 5 vs. 1	A, BMI	6081	USA	Both, breakdown not reported	FFDM	Brandt 2016 [61]
				OR 1.94 (1.48, 2.54)	BI-RADS-like category (4 vs. 2)						
				OR 1.3 (95% CI 1.1, 1.4)	per SD increase	A, BMI	1662	USA	Not reported	FFDM	Brandt 2016 [141]
				OR 1.51 (95% CI 1.12, 2.02) univariate analysis; OR 1.67 (95% CI 1.12, 2.27) multivariate analysis; screen-detected cancer	Quartile 4 vs. 1	None	1296	UK	Not reported	FFDM	Evans 2016 [34]
				OR 0.91 (95% CI 0.62, 1.33); future cancer	Quartile 4 vs. 1	When adjusted: A, BMI, M	33,142	UK	Not reported	FFDM	Evans 2016 [34]
			Volpara	RR 2.7	Octile 8 vs. 1	None specified	1158	Canada	Not reported	FFDM	Yaffe 2011 [143]
				OR 1.53 (95% CI 0.91, 2.68)	BI-RADS-like category (4 vs. 1)	A	33,029	Netherlands	Not reported	FFDM	Kallenberg 2012 [143]
				OR 8.26 (95% CI 4.28, 15.96)	Quintile 5 vs. 1	A, BMI, M, P	1099	UK	86.4%	FFDM	Eng 2014 [114]
				OR 1.83 (95% CI 1.51, 2.21)	per SD increase						
				OR 2.05 (95% CI 0.99,4.23) premenopausal; OR 3.07 (95% CI 1.89, 4.99) postmenopausal	BI-RADS-like category (4 vs. 2 and 1)	A, BMI, HRT, P	1984	South Korea	58.3%	FFDM	Park 2014 [144]
				OR 2.96 (95% CI 1.78, 4.93); screen-detected cancer	Quintile 5 vs. 1	None specified	1464	UK	Not reported	FFDM	Astley 2016 [122]
				OR 4.04 (95% CI 2.33, 7.01); future cancer	Quintile 5 vs. 1	None specified	1352	UK	Not reported	FFDM	Astley 2016 [122]
				HR 2.2 (95% CI 1.2, 4.1)	Quartile 4 vs. 1	None specified	5746	USA	Not reported	FFDM	Battle 2016 [145]
				OR 2.03 (95% CI 1.64, 2.51)	Quintile 5 vs. 2	A, BMI	6081	USA	Both, breakdown not reported	FFDM	Brandt 2016 [61]
				OR 1.82 (1.49, 2.21)	BI-RADS-like category (4 vs. 2)						
				OR 1.4 (95% CI 1.2, 1.6)	per SD increase	A, BMI	1662	USA	Not reported	FFDM	Brandt 2016 [141]
				OR 6.91 (95% CI 3.67, 13.04) raw images	Quintile 5 vs. 1	A, BMI, HRT, M, Men, OC, P	1098	UK	86.3%	FFDM	Busana 2016 [121]
				OR 1.20 (95% CI 0.92, 1.58) univariate analysis; OR 1.60 (95% CI 1.15, 2.23) multivariate analysis; screen-detected cancer	Quartile 4 vs. 1	None	1296	UK	Not reported	FFDM	Evans 2016 [34]
				OR 2.33 (95% CI 1.46, 3.72); future cancer	Quartile 4 vs. 1	When adjusted: A, BMI, M	33,142	UK	Not reported	FFDM	Evans 2016 [34]
				OR 1.71 (95% CI 0.83, 3.53)	Quartile 4 vs. 2	BMI, M, P	399	USA	67.2%	FFDM	Jeffers 2016 [124]
				OR 2.05 (95% CI 0.90, 6.64)	BI-RADS-like category (4 vs. 2)						

Abbreviations: A, age; BMI, body mass index; CI, confidence intervals; FB, age at first birth; FH, family history of breast cancer; HR, hazard ratio; HRT, use of hormone replacement therapy; M, menopausal status; Men, age at menarche; OC, oral contraceptive use; OR, odds ratio; P, parity; PrevBiop, number of previous biopsies; R, race; RR, relative risk.

## References

[1] Martin L.J., Boyd N.F. (2008). Mammographic density. Potential mechanisms of breast cancer risk associated with mammographic density: Hypotheses based on epidemiological evidence. Breast Cancer Res..

[2] Boyd N.F., Dite G.S., Stone J., Gunasekara A., English D.R., McCredie M.R., Giles G.G., Tritchler D., Chiarelli A., Yaffe M.J. (2002). Heritability of Mammographic Density, a Risk Factor for Breast Cancer. N. Engl. J. Med..

[3] Brand J.S., Humphreys K., Thompson D.J., Li J., Eriksson M., Hall P., Czene K. (2014). Volumetric Mammographic Density: Heritability and Association With Breast Cancer Susceptibility Loci. J. Natl. Cancer Inst..

[4] Stone J., Dite G.S., Gunasekara A., English D.R., McCredie M.R.E., Giles G.G., Cawson J.N., Hegele R.A., Chiarelli A.M., Yaffe M.J. (2006). The Heritability of Mammographically Dense and Nondense Breast Tissue. Cancer Edpidemiol. Biomark. Prev..

[5] Destounis S., Johnston L., Highnam R., Arieno A., Morgan R., Chan A. (2017). Using Volumetric Breast Density to Quantify the Potential Masking Risk of Mammographic Density. Am. J. Roentgenol..

[6] Boyd N.F., Guo H., Martin L.J., Sun L., Stone J., Fishell E., Jong R.A., Hislop G., Chiarelli A., Minkin S. (2007). Mammographic density and the risk and detection of breast cancer. N. Engl. J. Med..

[7] Yankaskas B.C., Cleveland R.J., Schell M.J., Kozar R. (2001). Association of Recall Rates with Sensitivity and Positive Predictive Values of Screening Mammography. Am. J. Roentgenol..

[8] Kerlikowske K. (2007). The mammogram that cried Wolfe. N. Engl. J. Med..

[9] Mandelson M.T., Oestreicher N., Porter P.L., White D., Finder C.A., Taplin S.H., White E. (2000). Breast density as a predictor of mammographic detection: Comparison of interval- and screen-detected cancers. J. Natl. Cancer Inst..

[10] Carney P.A., Miglioretti D.L., Yankaskas B.C., Kerlikowske K., Rosenberg R., Rutter C.M., Geller B.M., Abraham L.A., Taplin S.H., Dignan M. (2003). Individual and combined effects of age, breast density, and hormone replacement therapy use on the accuracy of screening mammography. Ann. Intern. Med..

[11] Kolb T.M., Lichy J., Newhouse J.H. (2002). Comparison of the performance of screening mammography, physical examination, and breast US and evaluation of factors that influence them: An analysis of 27,825 patient evaluations. Radiology.

[12] Park C.C., Remberg J., Chew K., Moore D., Kerliwkowske K. (2009). High mammographic breast density is independent predictor of local but not distant recurrence after lumpectomy and radiotherapy for invasive breast cancer. Int. J. Radiat. Oncol. Biol. Phys..

[13] Eriksson L., Czene K., Rosenberg L.U., Tornberg S., Humphreys K., Hall P. (2013). Mammographic density and survival in interval breast cancers. Breast Cancer Res..

[14] Ng K.-H., Lau S. (2015). Vision 20/20: Mammographic breast density and its clinical applications. Med. Phys..

[15] He W., Juette A., Denton E.R.E., Oliver A., Martí R., Zwiggelaar R. (2015). A review on automatic mammographic density and parenchymal segmentation. Int. J. Breast Cancer.

[16] Ekpo E.U., McEntee M.F. (2014). Measurement of breast density with digital breast tomosynthesis—A systematic review. Br. J. Radiol..

[17] Ekpo E.U., Hogg P., Highnam R., McEntee M.F. (2015). Breast composition: Measurement and clinical use. Radiography.

[18] Wolfe J.N. (1976). Breast patterns as an index of risk for developing breast cancer. Am. J. Roentgenol..

[19] Wolfe J.N. (1976). Risk for breast cancer development determined by mammographic parenchymal pattern. Cancer.

[20] Gram I.T., Funkhouser E., Tabar L. (1997). The Tabar classification of mammographic parenchymal patterns. Eur. J. Radiol..

[21] Egan R.L., Mosteller R.C. (1977). Breast cancer mammography patterns. Cancer.

[22] Mendell L., Rosenbloom M., Naimark A. (1977). Are breast patterns a risk index for breast cancer? A reappraisal. Am. J. Roentgenol..

[23] Ernster V.L., Sacks S.T., Peterson C.A., Schweitzer J.J. (1980). Mammographic parenchymal patterns and risk factors for breast cancer. Radiology.

[24] Moskowitz M., Gartside P., McLaughlin C. (1980). Mammographic patterns as markers for high-risk, benign breast disease and incident cancers. Radiology.

[25] McSweeney M.B. (1978). Breast parenchymal pattern as an indicator of risk for developing breast cancer. J. Med. Assoc. Ga..

[26] Brisson J., Diorio C., Masse B. (2003). Wolfe’s parenchymal pattern and percentage of the breast with mammographic densities: Redundant or complementary classifications?. Cancer Epidemiol. Biomark. Prev..

[27] Whitehead J., Carlile T., Kopecky K.J., Thompson D.J., Gilbert F.I., Present A.J., Threatt B.A., Krook P., Hadaway E. (1985). The Relationship between Wolfe’s Classification of Mammograms, Accepted Breast Cancer Risk Factors, and the Incidence of Breast Cancer. Am. J. Epidemiol..

[28] Boyd N.F., Jensen H.M., Cooke G., Han H.L. (1992). Relationship Between Mammographic and Histological Risk Factors for Breast Cancer. J. Natl. Cancer Inst..

[29] Boyd N.F., Byng J.W., Jong R.A., Fishell E.K., Little L.E., Miller A.B., Lockwood G.A., Tritchler D.L., Yaffe M.J. (1995). Quantitative classification of mammographic densities and breast cancer risk: Results from the Canadian National Breast Screening Study. J. Natl. Cancer Inst..

[30] Sergeant J.C., Warwick J., Evans D.G., Howell A., Berks M., Stavrinos P., Sahin S., Wilson M., Hufton A., Buchan I., Maidment A.D.A., Bakic P.R., Gavenonis S. (2012). Volumetric and Area-Based Breast Density Measurement in the Predicting Risk of Cancer at Screening (PROCAS) Study. IWDM 2012 Breast Imaging.

[31] Sukha A., Berks M., Morris J., Boggis C., Wilson M., Barr N., Astley S., Marti J., Oliver A., Freixenet J., Marti R. (2010). Visual Assessment of Density in Digital Mammograms. IWDM 2010 Digital Mammography.

[32] Sperrin M., Bardwell L., Sergeant J.C., Astley S., Buchan I. (2013). Correcting for rater bias in scores on a continuous scale, with application to breast density. Stat. Med..

[33] Beattie L., Harkness E., Bydder M., Sergeant J., Maxwell A., Barr N., Beetles U., Coggis C., Blundred S., Gadde S., Fujita H., Hara T., Muramatsu C. (2014). Factors Affecting Agreement between Breast Density Assessment Using Volumetric Methods and Visual Analogue Scales. IWDM 2014 Breast Imaging.

[34] Evans D.G., Astley S., Stavrinos P., Harkness E., Donnelly L.S., Dawe S., Jacob I., Harvie M., Cuzick J., Brentnall A. (2016). Improvement in Risk Prediction, Early Detection and Prevention of Breast Cancer in the NHS Breast Screening Programme and Family History Clinics: A Dual Cohort Study. Programme Grants for Applied Research.

[35] American College of Radiology (2003). BI-RADS^®^ Atlas.

[36] American College of Radiology (2013). BI-RADS^®^ Atlas.

[37] Byng J.W., Boyd N.F., Fishell E., Jong R.A., Yaffe M.J. (1994). The quantitative analysis of mammographic densities. Phys. Med. Biol..

[38] Harvey J.A., Bovbjerg V.E. (2004). Quantitative assessment of mammographic breast density: relationship with breast cancer risk. Radiology.

[39] Boyd N.F., Lockwood G.A., Byng J.W., Tritchler D.L., Yaffe M.J. (1998). Mammographic densities and breast cancer risk. Cancer Epidemiol. Biomark. Prev..

[40] Bakic P.R., Kontos D., Zhang C., Yaffe M.J., Maidment A.D.A., Giger M.L., Karssemeijer N. Analysis of percent density estimates from digital breast tomosynthesis projection images. Proceedings of the Medical Imaging 2007: Computer-Aided Diagnosis.

[41] Ursin G., Astrahan M.A., Salane M., Parisky Y.R., Pearce J.G., Daniels J.R., Pike M.C., Spicer D.V. (1998). The detection of changes in mammographic densities. Cancer Epidemiol. Biomark. Prev..

[42] Nguyen T.L., Aung Y.K., Evans C.F., Yoon-Ho C., Jenkins M.A., Sung J., Hopper J.L., Song Y.-M. (2015). Mammographic density defined by higher than conventional brightness threshold better predicts breast cancer risk for full-field digital mammograms. Breast Cancer Res..

[43] Li J., Szekely L., Eriksson L., Heddson B., Sundbom A., Czene K., Hall P., Humphreys K. (2012). High-throughput mammographic-density measurement: A tool for risk prediction of breast cancer. Breast Cancer Res..

[44] Keller B.M., Chen J., Daye D., Conant E.F., Kontos D. (2015). Preliminary evaluation of the publicly available Laboratory for Breast Radiodensity Assessment (LIBRA) software tool: Comparison of fully automated area and volumetric density measures in a case-control study with digital mammography. Breast Cancer Res..

[45] Eriksson M., Czene K., Pawitan Y., Leifland K., Darabi H., Hall P. (2017). A clinical model for identifying the short-term risk of breast cancer. Breast Cancer Res..

[46] Tagliafico A., Tagliafico G., Tosto S., Chiesa F., Martinoli C., Derchi L.E., Calabrese M. (2009). Mammographic density estimation: Comparison among BI-RADS categories, a semi-automated software and a fully automated one. Breast.

[47] iCad Inc. 2016 iReveal Product Brochure. http://www.icadmed.com/assets/dmm211_powerlook_density_assessment_reve.pdf.

[48] (2017). FDA, M-VU BREAST DENSITY 510(k) Premarket Notification. http://www.accessdata.fda.gov/cdrh_docs/pdf13/K132742.pdf.

[49] Keating D.M., D’Alessio D.D., Feigin K.N. BI-RADS 5th Edition Breast Density Classification: Comparison of Radiologist and Automated System. Proceedings of the RSNA 2015, Radiological Society of North America.

[50] Wengert G.J., Woitek R., Baltzer P., Kapetas P., Magometschnigg H., Bickel H., Helbich K., Pinker-Domenig K. Validation of a new fully automated quantitative breast density measuremet system with FFDM-comparison with qualitative ACR BI-RADS assessmet. Proceedings of the ECR 2015, European Congress of Radiology.

[51] Abdolell M., Tsuruda K.M., Lightfoot C.B., Payne J.I., Caines J.S., Iles S.E. (2015). Utility of relative and absolute measures of mammographic density vs clinical risk factors in evaluating breast cancer risk at time of screening mammography. Br. J. Radiol..

[52] Shepherd J.A., Herve L., Landau J., Fan B., Kerlikowske K., Cummings S.R. (2005). Novel use of Single X-ray Absorptiometry for Measuring Breast Density. Technol. Cancer Res. Treat..

[53] Alonzo-Proulx O., Packard N., Boone J.M., Al-Mayah A., Brock K.K., Shen S.Z., Yaffe M.J. (2010). Validation of a method for measuring the volumetric breast density from digital mammograms. Phys. Med. Biol..

[54] Alonzo-Proulx O., Jong R.A., Yaffe M.J. (2012). Volumetric breast density characteristics as determined from digital mammograms. Phys. Med. Biol..

[55] Hologic Inc. (2012). Understanding Quantra™ 2.0 User Manual.

[56] Van Engeland S., Snoeren P.R., Huisman H., Boetes C., Karssemeijer N. (2006). Volumetric breast density estimation from full-field digital mammograms. IEEE Trans. Med. Imaging.

[57] Highnam R., Brady M., Yaffe M.J., Karssemeijer N., Harvey J., Martí J., Oliver A., Freixenet J., Martí R. (2010). Robust breast composition measurement—Volpara™. Proceedings of the Digital Mammography: 10th International Workshop, IWDM 2010.

[58] Lau S., Ng K.-H., Abdul Aziz Y.F. (2016). Volumetric breast density measurement: Sensitivity analysis of a relative physics approach. Br. J. Radiol..

[59] Gweon H.M., Youk J.H., Kim J.A., Son E.J. (2013). Radiologist assessment of breast density by BI-RADS categories versus fully automated volumetric assessment. Am. J. Roentgenol..

[60] Seo J.M., Ko E.S., Han B.K., Ko E.Y., Shin J.H., Hahn S.Y. (2013). Automated volumetric breast density estimation: A comparison with visual assessment. Clin. Radiol..

[61] Brandt K.R., Scott C.G., Ma L., Mahmoudzadeh A.P., Jensen M.R., Whaley D.H., Wu F.F., Malkov S., Hruska C.B., Norman A.D. (2016). Comparison of clinical and automated breast density measurements: Implications for risk prediction and supplemental screening. Radiology.

[62] Wang J., Azziz A., Fan B., Malkov S., Klifa C., Newitt D., Yitta S., Hylton N., Kerlikowske K., Shepherd J.A. (2013). Agreement of mammographic measures of volumetric breast density to MRI. PLoS ONE.

[63] Gubern-Merida A., Kallenberg M., Platel B., Mann R.M., Marti R., Karssemeijer N. (2014). Volumetric breast density estimation from full field digital mammograms: A Validation Study. PLoS ONE.

[64] Ducote J.L., Molloi S. (2008). Quantification of breast density with dual energy mammography: A simulation study. Med. Phys..

[65] Koninklijke Philips N.V. (2015). Spectral Breast Density Measurement Tool Product Brochure. http://incenter.medical.philips.com/doclib/enc/11509347/4522_991_10201_Breast_Density_Tool_Product_Overview_Global_with_US_Final.pdf%3ffunc%3ddoc.Fetch%26nodeid%3d11509347.

[66] Kilburn-Toppin F., Erhard K., Willsher P., Buelow T., Wieberneit N., Fredenberg E., Wallis M.G. Characterisation of Breast Lesions with Spectral Mammography: Results on First Clinical Cases. Proceedings of the ECR 2015, European Congress on Radiology.

[67] Machida Y., Tozaki M., Yoshida T., Saita A., Yakabe M., Nii K. (2014). Feasibility study of a breast density measurement within a direct photon-counting mammography scanner system. Jpn. J. Radiol..

[68] Molloi S., Ding H., Feig S. (2015). Breast density evaluation using spectral mammography, radiologist reader assessment, and segmentation techniques: A retrospective study based on left and right breast comparison. Acad. Radiol..

[69] Ciatto S., Houssami N., Apruzzese A., Bassetti E., Brancato B., Carozzi F., Catarzi S., Lamberini M.P., Marcelli G., Pellizzoni R. (2005). Categorizing breast mammographic density: Intra- and interobserver reproducibility of BI-RADS density categories. Breast.

[70] Irshad A., Leddy R., Ackerman S., Cluver A., Pavic D., Abid A., Lewis M.C. (2016). Effects of changes in BI-RADS density assessment guidelines (fourth versus fifth edition) on breast density assessment: Intra- and interreader agreements and density distribution. Am. J. Roentgenol..

[71] Van der Waal D., den Heeten G.J., Pijnappel R.M., Schuur K.H., Timmers J.M.H., Verbeek A.L.M., Broeders M.J.M. (2015). Comparing visually assessed BI-RADS breast density and automated volumetric breast density software: A cross-sectional study in a breast cancer screening setting. PLoS ONE.

[72] Garrido-Estepa M., Ruiz-Perales F., Miranda J., Ascunce N., González-Román I., Sánchez-Contador C., Santamarina C., Moreo P., Vidal C., Peris M. (2010). Evaluation of mammographic density patterns: Reproducibility and concordance among scales. BMC Cancer.

[73] Sprague B.L., Conant E.F., Onega T., Garcia M.P., Beaber E.F., Herschorn S.D., Lehman C.D., Tosteson A.N.A., Lacson R., Schnall M.D. (2016). Variation in mammographic breast density assessments among radiologists in clinical practice: A multicenter observational study. Ann. Intern. Med..

[74] Raza S., Mackesy M.M., Winkler N.S., Hurwitz S., Birdwell R.L. (2016). Effect of training on qualitative mammographic density assessment. J. Am. Coll. Radiol..

[75] Youk J.H., Gweon H.M., Son E.J., Kim J.A. (2016). Automated volumetric breast density measurements in the era of the BI-RADS fifth edition: A comparison with visual assessment. Am. J. Roentgenol..

[76] Conant E.F., Li D., Gavenonis S., Bakic P.R., Carton A.-K., Zhang C., Maidment A.D.A., Kontos D. (2010). A Comparative Study of the Inter-Reader Variability of Breast Percent Density Estimation in Digital Mammography: Potential Effect of Reader’s Training and Clinical Experience. IWDM 2010 Digital Mammography.

[77] Alonzo-Proulx O., Mawdsley G.E., Patrie J.T., Yaffe M.J., Harvey J.A. (2015). Reliability of Automated Breast Density Measurements. Radiology.

[78] Zanca F., Jacobs J., van Ongeval C., Claus F., Celis V., Geniets C., Provost V., Pauwels H., Marchal G., Bosmans H. (2009). Evaluation of clinical image processing algorithms used in digital mammography. Med. Phys..

[79] Burton A., Byrnes G., Stone J., Tamimi R.M., Heine J., Vachon C., Ozmen V., Pereira A., Garmendia M.L., Scott C. (2016). Mammographic density assessed on paired raw and processed digital images and on paired screen-film and digital images across three mammography systems. Breast Cancer Res..

[80] Vinnicombe S.J., Evans A.J., Hart K., Whelehan P., Dundee G.B. Visual And Automated Volumetric Assessment of Mammographic Density (MD): Do Measurements Depend on the Digital Mammography Unit?. Proceedings of the ECR 2014, European Congress on Radiology.

[81] Damases C.N., Brennan P.C., McEntee M.F. (2015). Mammographic density measurements are not affected by mammography system. J. Med. Imaging.

[82] Skaane P., Bandos A.I., Eben E.B., Jebsen I.N., Krager M., Haakenaasen U., Ekseth U., Izadi M., Hofvind S., Gullien R. (2014). Two-view digital breast tomosynthesis screening with synthetically reconstructed projection images: Comparison with digital breast tomosynthesis with full-field digital mammographic images. Radiology.

[83] Svahn T.M., Houssami N., Sechopoulos I., Mattsson S. (2015). Review of radiation dose estimates in digital breast tomosynthesis relative to those in two-view full-field digital mammography. Breast.

[84] Martínez-Miravete P., Millor Muruzábal M., García-Barquín P., Elizalde A., Pina L., Etxano J., Bartolomé P. Does the Synthesised Digital Mammography (3D-DM) Change the ACR Density Pattern?. Proceedings of the ECR 2015, European Congress of Radiology.

[85] Conant E.F., Keller B.M., Pantalone L., Gastounioti A., McDonald E.S., Kontos D. (2017). Agreement between Breast Percentage Density Estimattions from Standard-Dose versus Synthetic Digital Mammograms: Results from a Large Screening Cohort Using Autimated Measures. Radiology.

[86] Pertuz S., McDonald E.S., Weinstein S.P., Conant E.F., Kontos D. (2016). Fully automated quantitative estimation of volumetric breast density from digital breast tomosynthesis images: Preliminary results and comparison with digital mammography and MR imaging. Radiology.

[87] Tromans C., Highnam R., Morrish O., Black R., Tuckers L., Gilbert F.J. Volumetric Breast Density Estimation on Conventional Mammography Versus Digital Breast Tomosynthesis. Proceedings of the ECR 2014, European Congress of Radiology.

[88] Pisano E.D., Hendrick R.E., Yaffe M.J., Baum J.K., Acharyya S., Cormack J.B., Hanna L.A., Conant E.F., Fajardo L.L., Bassett L.W. (2008). Diagnostic Accuracy of Digital versus Film Mammography: Exploratory Analysis of Selected Population Subgroups in DMIST. Radiology.

[89] Prummel M.V., Muradali D., Shumak R., Majpruz V., Brown P., Jiang H., Done S.J., Yaffe M.J., Chiarelli A.M. (2016). Digital Compared with Screen-Film Mammography: Measures of Diagnostic Accuracy among Women Screened in the Ontario Breast Screening Program. Radiology.

[90] Wanders J.O., Holland K., Veldhuis W.B., Mann R.M., Pijnappel R.M., Peeters P.H., van Gils C.H., Karssemeijer N. (2017). Volumetric breast density affects performance of digital screening mammography. Breast Cancer Res. Treat..

[91] Maniprize J.G., Alonzo-Proulx O., Jon R.A., Yaffe M.J. (2016). Quantifying masking in clinial mammograms via local detectability of simulated lesions. Med. Phys..

[92] Holland K., van Zelst J., den Heeten G.J., Imhof-Tas M., Mann R.M., van Gils C.H., Karssemeijer N. (2016). Consistency of breast density categories in serial screening mammograms: A comparison between automated and human assessment. Breast.

[93] Are You Dense Inc. Are You Dense? Exposing the Best-Kept Secret. https://www.areyoudense.org.

[94] Freer P.E. (2015). Mammographic breast density: Impact on breast cancer risk and implications for screening. Radiographics.

[95] DenseBreast-Info Inc. DenseBreast-Info. http://densebreast-info.org/.

[96] Breast Density and Mammography Reporting Act of 2015, H.R.716. Proceedings of the 114th Congress (2015).

[97] Schilling K., The J., Griff S., Oliver L., Mahal R., Saady M., Velasquez V. Impact of quantitative breast density on experienced radiologists’ assessment of mammographic breast density. Proceedings of the ECR 2015, European Congress of Radiology.

[98] Berg W.A., Blume J.D., Cormack J.B., Mendelson E.B., Lehrer D., Bohm-Velez M., Pisano E.D., Jong R.A., Evans W.P., Morton M.J. (2008). Combined Screening With Ultrasound and Mammography vs. Mammography Alone in Women at Elevated Risk of Breast Cancer. JAMA.

[99] Berg W.A., Zhang Z., Lehrer D., Jong R.A., Pisano E.D., Barr R.G., Bohm-Velez M., Mahoney M.C., Evans W.P., Larsen L.H. (2012). Detection of Breast Cancer With Addition of Annual Screening Ultrasound or a Single Screening MRI to Mammography in Women with Elevated Breast Cancer Risk. JAMA.

[100] FDA, PMA Notification Letter. https://google2.fda.gov/search?q=cache:SKN1t-KxTD0J:www.accessdata.fda.gov/cdrh_docs/pdf5/k052355.pdf+U-Systems&client=FDAgov&site=FDAgov&lr=&proxystylesheet=FDAgov&output=xml_no_dtd&ie=UTF-8&access=p&oe=UTF-8.

[101] Wilczek B., Wilczek H.E., Rasouliyan L., Leifland K. (2016). Adding 3D automated breast ultrasound to mammography screening in women with heterogeneously and extremely dense breasts: Report from a hospital-based, high-volume, single-center breast cancer screening program. Eur. J. Radiol..

[102] Friedewald S.M., Rafferty E.A., Rose S.L., Durand M.A., Plecha D.M., Greenberg J.S., Hayes M.K., Copit D.S., Carlson K.L., Cink T.M. (2014). Breast cancer screening using tomosynthesis in combination with digital mammography. JAMA.

[103] Ciatto S., Houssami N., Bernardi D., Caumo F., Pellegrini M., Brunelli S., Tuttobene P., Bricolo P., Fanto C., Valentini M. (2013). Integration of 3D digital mammography with tomosynthesis for population breast-cancer screening (STORM): A prospective comparison study. Lancet Oncol..

[104] Destounis S.V., Morgan R., Arieno A. (2015). Screening for Dense Breasts: Digital Breast Tomosynthesis. Am. J. Roentgenol..

[105] Tagliafico A.S., Calabrese M., Mariscotti G., Durando M., Tosto S., Monetti F., Airaldi S., Bignotti B., Nori J., Bagni A. (2016). Adjunct Screening with Tomosynthesis or Ultrasound in Women with Mammography-Negative Dense Breasts: Interim Report of a Prospective Comparative Trial. J. Clin. Oncol..

[106] Melnikow J., Fenton J.J., Whitlock E.P., Miglioretti D.L., Weyrich M.S., Thompson J.H., Shah K. (2016). Supplemental screening for breast cancer in women with dense breasts: A systematic review for the U.S. Preventive Services Task Force. Ann. Intern. Med..

[107] Shermis R.B., Wilson K.D., Doyle M.T., Martin T.S., Merryman D., Kudrolli H., Brenner R.J. (2016). Supplemental breast cancer screening with molecular breast imaging for women with dense breast tissue. Am. J. Roentgenol..

[108] Emaus M.J., Bakker M.F., Peeters P.H., Loo C.E., Mann R.M., de Jong M.D., Bisschops R.H., Veltman J., Duvivier K.M., Lobbes M.B. (2015). MR Imaging as an Additional Screening Modality for the Detection of Breast Cancer in Women Aged 50–75 Years with Extremely Dense Breasts: The DENSE Trial Study Design. Radiology.

[109] ClincalTrials.Gov. https://clinicaltrials.gov/ct2/show/NCT02933489.

[110] Destounis S., Arieno A., Morgan R. (2015). Initial Experience With the New York State Breast Density Inform Law at a Community-Based Breast Center. J. Ultrasound. Med..

[111] Mainiero M.B., Lourenco A., Mahoney M.C., Newell M.S., Bailey L., Barke L.D., D’Orsi C., Harvey J.A., Hayes M.K., Huynh P.T. (2013). ACR Appropriateness Criteria Breast Cancer Screening. J. Am. Coll. Radiol..

[112] Sprague B.L., Stout N.K., Schechter C., van Ravesteyn N.T., Cevik M., Alagoz O., Lee C.I., van den Broek J.J., Miglioretti D.L., Mandelblatt J.S. (2015). Potential impact of legislation mandating breast density notification: Benefits, harms, and cost effectiveness of supplemental utlrasound screening. Ann. Intern. Med..

[113] Plevritis S.K., Kurian A.W., Sigal B.M., Daniel B.L., Ikeda D.M., Stockdale F.E., Garber A.M. (2006). Cost-effectiveness of screening BRCA1/2 mutation carriers with breast magnetic resonance imaging. JAMA.

[114] Eng A., Gallant Z., Shepherd J., McCormack V., Li J., Dowsett M., Vinnicombe S., Steve A., dos-Santos-Silva I. (2014). Digital mammographic density and breast cancer risk: A case–control study of six alternative density assessment methods. Breast Cancer Res..

[115] Bertrand K.A., Tamimi R.M., Scott C.G., Jensen M.R., Pankratz V.S., Visscher D., Norman A., Couch F., Shepherd J., Fan B. (2013). Mammographic density and risk of breast cancer by age and tumor characteristics. Breast Cancer Res..

[116] American Cancer Society (2017). Cancer Facts & Figures 2017.

[117] Sprague B.L., Gangnon R.E., Burt V., Trentham-Dietz A., Hampton J.M., Wellman R.D., Kerlikowske K., Miglioretti D.L. (2014). Prevalence of mammographically dense breasts in the United States. J. Natl. Cancer Inst..

[118] Byrne C., Schairer C., Wolfe J., Parekh N., Salane M., Brinton L.A., Hoover R., Haile R. (1995). Mammographic features and breast cancer risk: Effects with time, age, and menopause status. J. Natl. Cancer Inst..

[119] Engmann N.J., Golmakani M.K., Miglioretti D.L., Sprague B.L., Kerlikowske K. (2017). Population-Attributable Risk Proportion of Clinical Risk Factors for Breast Cancer. JAMA Oncol..

[120] Yaffe M.J. (2008). Mammographic density. Measurement of mammographic density. Breast Cancer Res..

[121] Busana M.C., Eng A., Denholm R., Dowsett M., Vinnicombe S., Allen S., dos-Santos-Silva I. (2016). Impract of type of full-field digital image on mammographic density assessment and breast cancer risk estimation: A case-control study. Breast Cancer Res..

[122] Astley S., Harkness E., Sergeant J., Stavrinos P., Warren R., Wilson M., Brentnall A., Cuzick J., Howell A., Evans G. (2016). Proffered Paper: A Comparison of four methods of mammogrpahic density measurement in the UK Predicting Risk of Cancer at Screening (PROCAS) study-on behalf of the PROCAS Study team. Eur. J. Cancer.

[123] Sergeant J.C., Wilson M., Barr N., Beetles U., Boggis C., Bundred S., Bydder M., Gadde S., Hurley E., Jain A. (2013). PB.17: Inter-observer agreement in visual analogue scale asssessment of percentage breast density. Breast Cancer Res..

[124] Jeffers A.M., Sieh W., Lipson J.A., Rothstein J.H., McGuire V., Whittemore A.S., Rubin D.L. (2016). Breast Cancer Risk and Mammographic Density Assessed with Semiautomated and Fully Automated Methods and BI-RADS. Radiology.

[125] McCormack V.A., dos Santos Silva I. (2006). Breast density and parenchymal patterns as markers of breast cancer risk: A meta-analysis. Cancer Epidemiol Biomark. Prev..

[126] Shepherd J.A., Kerlikowske K., Ma L., Duewer F., Fan B., Wang J., Malkov S., Vittinghoff E., Cummings S.R. (2011). Volume of mammographic density and risk of breast cancer. Cancer Epidemiol. Biomark. Prev..

[127] Jakes R.W., Duffy S.W., Ng F.C., Gao F., Ng E.H. (2000). Mammographic parenchymal patterns and risk of breast cancer at and after a prevalence screen in Singapore women. Int. J. Epidemiol..

[128] Ziv E., Tice J., Smith-Bindman R., Shepherd J., Cummings S., Kerlikowske K. (2004). Mammographic Density and Estrogen Receptor Status of Breast Cancer. Cancer Epidemiol. Biomark. Prev..

[129] Barlow W.E., White E., Ballard-Barbash R., Vacek P.M., Titus-Ernstoff L., Carney P.A., Tice J.A., Buist D.S., Geller B.M., Rosenberg R. (2006). Prospective Breast Cancer Risk Prediction Model for Women Undergoing Screening Mammography. J. Natl. Cancer Inst..

[130] Olson A.H., Bihrmann K., Jensen M.B., Vejborg I., Lynge E. (2009). Breast density and outcome of mammography screening: A cohort study. Br. J. Cancer.

[131] Abdolell M., Tsuruda K., Payne J.I., Iles S.E., Lightfoot C.B., Caines J. Breast Density from Full-Field Digital Mammograms and Breast Cancer Risk: A Case-Control Study. Proceedings of the ECR 2014, European Congress of Radiology.

[132] Cuzick J., Warwick J., Pinney E., Duffy S.W., Cawthorn S., Howell A., Forbes J.F., Warren R.M. (2011). Tamoxifen-Induced Reduction in Mammographic Density and Breast Cancer Risk Reduction: A Nested Case-Control Study. J. Natl. Cancer Inst..

[133] Brentnall A.R., Harkness E.F., Astley S.M., Donnelly L.S., Stavrinos P., Sampson S., Fox L., Sergeant J.C., Harvie M.N., Wilson M. (2015). Mammographic density adds accuracy to both the Tyrer-Cuzick and Gail breast cancer risk models in a prospective UK screening cohort. Breast Cancer Res..

[134] Boyd N.F., Martin L.J., Sun L., Guo H., Chiarelli A., Hislop G., Yaffe M., Minkin S. (2006). Body size, mammographic density, and breast cancer risk. Cancer Epidemiol. Biomark. Prev..

[135] Aiken Z., McCormack V.A., Highnam R.P., Martin L., Gunasekara A., Melnichouk O., Mawdsley G., Peressotti C., Yaffe M., Boyd N.F. (2010). Screen-film mammographic density and breast cancer risk: A comparison of the volumetric standard mammogram form and the interactive threshold measurement methods. Cancer Epidemiol. Biomark. Prev..

[136] Lokate M., Peeters P.H., Peelen L.M., Haars G., Veldhuis W.B., Veldhuis W.B., van Gils C.H. (2011). Mammographic density and breast cancer risk: The role of the fat surrounding the fibroglandular tissue. Breast Cancer Res..

[137] Pettersson A., Hankinson S.E., Willett W.C., Lagiou P., Trichopoulos D., Tamimi R.M. (2011). Nondense mammographic area and risk of breast cancer. Breast Cancer Res..

[138] Nickson C., Arzhaeva Y., Aitken Z., Elgindy T., Buckley M., Li M., English D.R., Kavanagh A.M. (2013). AutoDensity: An automated method to measure mammographic breast density that predicts breast cancer risk and screening outcomes. Breast Cancer Res..

[139] Ursin G., Ma H., Wu A.H., Bernstein L., Salane M., Parisky Y.R., Astrahan M., Siozon C.C., Pike M.C. (2003). Mammographic density and breast cancer in three ethnic groups. Cancer Epidemiol. Biomark. Prev..

[140] Rauh C., Hack C.C., Haberle L., Hein A., Engel A., Schrauder M.G., Fasching P.A., Jud S.M., Ekici A.B., Loehberg C.R. (2012). Percent Mammographic Density and Dense Area as Risk Factors for Breast Cancer. Geburtshilife Frauenheilkd..

[141] Brandt K.R., Hsieh M.-K., Scott C.G., Pantalone L., Jensen M., Winham S., Whaley D.H., Hruska C.B., Wu F.F., Norman A. Validation Study of the Publically-Available Fully-Automated “LIBRA” Software for Mammographic Density Estimation: Results from a Case-Control Study of Breast Cancer. Proceedings of the RSNA 2016, Radiological Society of North America 2016 Scientific Assembly and Annual Meeting.

[142] Yaffe & Alonzo-Proulx Volumetric Breast Density and Breast Cancer Risk from Digital Mammograms—Preliminary Results. Proceedings of the 5th International Workshop on Breast Densitometry and Breast Cancer Risk Assessment.

[143] Kallenberg M., van Gils C., Mann R., Karssemeijer N. Association between Automated, Volumetric Measures of Breast Density and Diagnostic Outcome of Mammography Screening Examinations. Proceedings of the RSNA 2012, Radiological Society of North America 2012 Scientific Assembly and Annual Meeting.

[144] Park I.H., Ko K., Joo J., Park B., Jung S.Y., Lee S., Kwon Y., Kang H.S., Lee E.S., Lee K.S. (2014). High volumetric breast density predicts risk for breast cancer in postmenopausal, but not premenopausal, Korean Women. Ann. Surg. Oncol..

[145] Battle B., Malak S.F., Dhakal I., Lee J., Keith N., Fuhrman B. Associations of Volumetric Mammographic Density Measures with Breast Cancer Risk in 5746 Women. Proceedings of the RSNA 2016, Radiological Society of North America 2016 Scientific Assembly and Annual Meeting.

[146] Tice J.A., Cummings S.R., Ziv E., Kerlikowske K. (2005). Mammographic breast density and the Gail model for breast cancer risk prediction in a screening population. Breast Cancer Res. Treat..

[147] Tice J.A., Cummings S.R., Smith-Bindman R., Ichikawa L., Barlow W.E., Kerlikowske K. (2008). Using clinical factors and mammographic breast density to estimate breast cancer risk: Development and validation of a new predictive model. Ann. Intern. Med..

[148] (2016). NCCN, Breast Cancer Risk Reduction. https://www.nccn.org/professionals/physician_gls/pdf/breast_risk.pdf.

[149] Smith R.A., Andrews K., Brooks D., DeSantis C.E., Fedewa S.A., Lortet-Tieulent J., Manassaram-Baptiste D., Brawley O.W., Wender R.C. (2016). Cancer screening in the United States, 2016: A review of current American Cancer Society guidelines and current issues in cancer screening. CA Cancer J. Clin..

[150] (2016). USPSTF, Medications for Risk Reduction of Primary Breast Cancer in Women: U.S. Preventive Services Task Force Recommendation Statement. https://www.uspreventiveservicestaskforce.org/Page/Document/RecommendationStatementFinal/breast-cancer-medications-for-risk-reduction.

[151] Cuzick J. IBIS Breast Cancer Risk Evaluation Tool. http://www.ems-trials.org/riskevaluator/.

[152] Ekpo E.U., Brennan P.C., Mello-Thoms C., McEntee M.F. (2016). Relationship between breast density and selective estrogen-receptor modulators, aromatase inhibitors, physical activity, and diet: A systematic review. Integr. Cancer Ther..

[153] Habel L.A., Capra A.M., Achacoso N.S., Janga A., Acton L., Puligandla B., Quesenberrgy C.P. (2004). Mammographic Density and Risk of Second Breast Cancer After Ductal Carcinoma in situ. J. Natl. Cancer Inst..

[154] Cil T., Fishell E., Hanna W., Sun P., Rawlinson E., Narod S.A., McCready D.R. (2009). Mammographic Density and the Risk of Breast Cancer Recurrence After Breast-Conserving Surgery. Cancer.

[155] Chiu S.Y., Duffy S., Yen A.M., Tabar L., Smith R.A., Chen H.H. (2010). Effect of baseline breast density on breast cancer incidence, stage, mortality, and screening parameters: 25-Year follow-up of a Swedish mammographic screening. Cancer Epidemiol. Biomark. Prev..

[156] Porter G.J.R., Evans A.J., Cornford E.J., Burrell H.C., James J.J., Lee A.H.S., Charkrabarti J. (2007). Influence of Mammographic Parenchymal Pattern in Screening-Detected and Interval Invasive Breast Cancers on Pathologic Features, Mammographic Features, and Patient Survival. Am. J. Roentgenol..

[157] Gierach G.L., Ichikawa L., Kerlikowske K., Brinton L.A., Farhat G.N., Vacek P.M., Weaver D.L., Schairer C., Taplin S.H., Sherman M.E. (2012). Relationship Between Mammographic Density and Breast Cancer Death in the Breast Cancer Surveillance Consortium. J. Natl. Cancer Inst..

[158] Holm J., Humphreys K., Li J., Ploner A., Cheddad A., Eriksson M., Tornberg S., Hall P., Czene K. (2015). Risk factors and tumor characteristics of interval cancers by mammographic density. J. Clin. Oncol..

[159] Maskarinec G., Pagano I.S., Little M.A., Conroy S.M., Park S.-Y., Kolonel L.N. (2013). Mammographic density as a predictor of breast cancer survival: The Multiethnic Cohort. Breast Cancer Res..

[160] Ghosh K., Hartmann L.C., Reynolds C., Visscher D.W., Brandt K.R., Vierkant R.A., Scott C.G., Radisky D.C., Sellers T.A., Pankratz V.S. (2010). Association Between Mammographic Density and Age-Related Lobular Involution of the Breast. J. Clin. Oncol..

[161] McCormack V.A., Perry N.M., Vinnicombe S.J., dos Santos Silva I. (2010). Changes and tracking of mammographic density in relation to Pike’s model of breast tissue aging: A UK longitudinal study. Int. J. Cancer.

[162] Kelemen L.E., Pankratz V.S., Sellers T.A., Brandt K.R., Wang A., Janney C., Fredericksen Z.S., Cerhan J.R., Vachon C.M. (2008). Age-specific trends in mammographic density: The Minnesota Breast Cancer Family Study. Am. J. Epidemiol..

[163] Greendale G.A., Reboussin B.A., Slone S., Wasilauskas C., Pike M.C., Ursin G. (2003). Postmenopausal hormone therapy and change in mammographic density. J. Natl. Cancer Inst..

[164] Maskarinec G., Pagano I., Lurie G., Kolonel L.N. (2006). A longitudinal investigation of mammographic density: The multiethnic cohort. Cancer Epidemiol. Biomark. Prev..

[165] McTiernan A., Martin C.F., Peck J.D., Aragaki A.K., Chlebowski R.T., Pisano E.D., Wang C.Y., Brunner R.L., Johnson K.C., Manson J.E. (2005). Estrogen-plus-progestin use and mammographic density in postmenopausal women: Women’s Health Initiative randomized trial. J. Natl. Cancer Inst..

[166] Byrne C., Ursin G., Martin C.F., Peck J.D., Cole E.B., Zeng D., Kim E., Yaffe M.D., Boyd N.F., Heiss G. (2017). Mammographic density change with estrogen and progestin therapy and breast cancer risk. J. Natl. Cancer Inst..

[167] Li J., Humphreys K., Eriksson L., Edgren G., Czene K., Hall P. (2013). Mammographic density reduction is a prognostic marker of response to adjuvant tamoxifen therapy in postmenopausal patients with breast cancer. J. Clin. Oncol..

[168] Busana M.C., De Stavola B.L., Sovio U., Li J., Moss S., Humphreys K., dos-Santos-Silva I. (2016). Assessing within-woman changes in mammographic density: A comparison of fully versus semi-automated area-based approaches. Cancer Causes Control.

[169] Hammann-Kloss J.S., Bick U., Fallenberg E., Engelken F. (2014). Volumetric quantification of the effect of aging and hormone replacement therapy on breast composition from digital mammograms. Eur. J. Radiol..

[170] Lokate M., Stellato R.K., Veldhuis W.B., Peeters P.H., van Gils C.H. (2013). Age-related changes in mammographic density and breast cancer risk. Am. J. Epidemiol..

[171] Vachon C.M., Pankratz V.S., Scott C.G., Maloney S.D., Ghosh K., Brandt K.R., Milanese T., Carston M.J., Sellers T.A. (2007). Longitudinal trends in mammographic percent density and breast cancer risk. Cancer Epidemiol. Biomark. Prev..

[172] Vachon C.M., Suman V.J., Brandt K.R., Kosel M.L., Buzdar A.U., Olson J.E., Wu F.F., Flickinger L.M., Ursin G., Elliott C.R. (2013). Mammographic breast density response to aromatase inhibition. Clin. Cancer Res..

[173] Meggiorini M.L., Labi L., Vestri A.R., Porfiri L.M., Savelli S., DeFelice C. (2008). Tamoxifen in women with breast cancer and mammographic density. Eur. J. Gynaecol. Oncol..

[174] Nyante S.J., Sherman M.E., Pfeiffer R.M., de Gonzalez A.B., Brinton L.A., Bowles E.J.A., Hoover R.N., Glass A., Gierach G.L. (2016). Longitudinal Change in Mammographic Density among ER-Positive Breast Cancer Patient Using Tamoxifen. Cancer Epidemiol. Biomark. Prev..

[175] Borgquist S., Eriksson M., Czene K., Hall P. Abstract OT3-06-01: KARISMA, The karma intervention study—A tamoxifen dose determination trial. Proceedings of the 2016 San Antonio Breast Cancer Symposium.

[176] Nyante S.J., Sherman M.E., Pfeiffer R.M., Berrington de Gonzalez A., Brinton L.A., Aiello Bowles E.J., Hoover R.N., Glass A., Gierach G.L. (2015). Prognostic Significance of Mammographic Density Change after Initiation of Tamoxifen for ER-Positive Breast Cancer. J. Natl. Cancer Inst..

[177] Ko K., Shin I., You J., Jung S.-Y., Ro J., Lee E.S. (2013). Adjuvant tamoxifen-induced mammographic breast density reduction as a predictor for recurrence in estrogen receptor-positive premenopausal breast cancer patients. Breast Cancer Res. Treat..

[178] Kim J., Wonshik H., Hyeong-Gon M., Ahn S.K., Shin H.-C., You J.-M., Han S.-W., Im S-A., Kim T.-Y., Koo H.R. (2012). Breast density change as a predictive surrogate for response to adjuvant endocrine therapy in hormone receptor positive breast cancer. Breast Cancer Res..

[179] Mourits M.J., de Vries E.G., Willemse P.H., Ten Hoor K.A., Hollema H., van der Zee A.G. (2001). Tamoxifen treatment and gynecologic side effects: A review. Obstet. Gynecol..

[180] Moyer V.A. (2013). Medications to decrease the risk for breast cancer in women: Recommendations from the U.S. Preventive Services Task Force recommendation statement. Ann. Intern. Med..

[181] Jordan V.C. (2007). New insights into the metabolism of tamoxifen and its role in the treatment and prevention of breast cancer. Steroids.

[182] Engmann N.J., Scott C., Jensen M.R., Ma L., Brandt K.R., Mahmoudzadeh A., Maikov S., Whaley D.H., Hruska C., Wu F.F. (2017). Longitudinal changes in volumetric breast density with tamoxifen and aromatase inhibitors. Cancer Epidemiol. Biomark. Prev..

[183] Shawky M.S., Martin H., Hugo H.J., Lloyd T., Britt K.L., Redfern A., Thompson E.W. (2017). Mammographic density: A potential monitoring biomarker for adjuvant and preventative breast cancer endocrine therapies. Oncotarget.

[184] Sestak I., Cuzick J. (2008). Breast cancer chemoprevention. Oncol. Rev..

[185] Guvakova M.A., Surmacz E. (1997). Tamoxifen interferes with the insulin-like growth factor I receptor (IGF-IR) signaling pathway in breast cancer cells. Cancer Res..

[186] Pollak M.N., Huynh H.T., Lefebvre S.P. (1992). Tamoxifen reduces serum insulin-like growth factor I (IGF-I). Breast Cancer Res. Treat..

[187] Diorio C., Pollak M., Byrne C., Masse B., Hebert-Croteau N., Yaffe M., Cote G., Berube S., Morin C., Brisson J. (2005). Insulin-like growth factor-I, IGF-binding protein-3, and mammographic breast density. Cancer Epidemiol. Biomark. Prev..

[188] Dos Santos Silva I., Johnson N., de Stavola B., Torres-Mejia G., Fletcher O., Allen D.S., Allen N.E., Key T.J., Fentiman I.S., Holly J.M.P. (2006). The insulin-like growth factor system and mammographic features in premenopausal and postmenopausal women. Cancer Epidemiol. Biomark. Prev..

[189] Huo C.W., Chew G.L., Britt K.L., Ingman W.V., Henderson M.A., Hopper J.L., Thompson E.W. (2014). Mammographic density—A review on the current understanding of its association with breast cancer. Breast Cancer Res. Treat..

[190] Sun X., Glynn D.J., Hodson L.J., Huo C., Britt K., Thompson E.W., Woolford L., Evdokiou A., Pollard J.W., Robertson S.A. (2017). CCL2-driven inflammation increases mammary gland stromal density and cancer susceptibility in a transgenic mouse model. Breast Cancer Res..

[191] Kerlikowske K., Zhu W., Tosteson A.N., Sprague B.L., Tice J.A., Lehman C.D., Miglioretti D.L. (2015). Identifying women with dense breats at high risk for interval cancer: A cohort study. Ann. Intern. Med..

